# Changes of gut microbiome composition and metabolites associated with hypertensive heart failure rats

**DOI:** 10.1186/s12866-021-02202-5

**Published:** 2021-05-05

**Authors:** Lin Li, Sen-jie Zhong, Si-yuan Hu, Bin Cheng, Hong Qiu, Zhi-xi Hu

**Affiliations:** 1 The Domestic First-class Discipline Construction Project of Chinese Medicine, Hunan University of Chinese Medicine, Changsha, Hunan China; 2 Institute of Traditional Chinese Medicine Diagnostics, Hunan University of Chinese Medicine, Changsha, Hunan China; 3 Post-Graduate School, Hunan University of Chinese Medicine, Changsha, Hunan China

**Keywords:** HF, Gut microbiota, Fecal metabolomics, 16S rRNA sequencing. Gut-heart axis

## Abstract

**Background:**

The potential role of the gut microbiome (GM) in heart failure (HF) had recently been revealed. However, the underlying mechanisms of the GM and fecal metabolome in HF have not been characterized. The Dahl salt-sensitive rat model of hypertensive heart failure (H-HF) was used to study the clinical symptoms and characteristics. To elucidate the pathogenesis of HF, we combined 16S rRNA gene sequencing and metabolomics to analyze gut microbial compositions and fecal metabolomic profiles of rats with H-HF.

**Results:**

PCoA of beta diversity shown that the gut microbiome composition profiles among the three groups were separated. Gut microbial composition was significantly altered in H-HF rats, the ratio of *Firmicutes* to *Bacteroidetes*(F/B) increased and the abundance of *Muribaculaceae*, *Lachnospiraceae*, and *Lactobacillaceae* decreased. Significantly altered levels of 17 genera and 35 metabolites were identified as the potential biomarker of H-HF. Correlation analysis revealed that specific altered genera were strongly correlated with changed fecal metabolites. The reduction in short-chain fatty acids (SCFA)-producing bacteria and trimethylamine N-oxide (TMAO) might be a notable characteristic for H-HF.

**Conclusions:**

This is the first study to characterize the fecal microbiome of hypertensive heart failure by integrating 16S rRNA gene sequencing and LC–MS-based metabolomics approaches. Collectively, the results suggesting changes of gut microbiome composition and metabolites are associated with hypertensive heart failure rats.

**Supplementary Information:**

The online version contains supplementary material available at 10.1186/s12866-021-02202-5.

## Background

Heart failure (HF) is the terminal stage of all cardiac diseases, with high morbidity and mortality rates [1]. Epidemiological data revealed that the prevalence of HF is 1–2% in adults and increases to more than 10% in people over the age of 70 [2]. The leading causes of HF are hypertension and ischemic heart diseases, and HF resulting from hypertension has become to be a major public health concern [3]. Therefore, timely diagnosis and early treatment are the keys. The gut microbiome (GM) is a complex community of trillions of bacteria in the gastrointestinal tract and has emerged as a central factor affecting human health and disease [4]. Host-microbiota interactions involving inflammatory and metabolic pathways have been linked to the pathogenesis of cardiovascular disease [5, 6]. A growing number of studies have shown that GM is closely related to the occurrence and development of HF, in addition to alterations in GM composition, the metabolic potential of GM has been identified as a contributing factor in the development of diseases, including the trimethylamine (TMA)/ trimethylamine N-oxide (TMAO) pathway, short-chain fatty acids (SCFA) pathway, bile acid pathway and uremic toxin pathways, so microbiota is expected to become an essential target for intervention of HF [7]. HF has long been recognized to be associated with changed in intestinal function [8]. The gut hypothesis suggests that HF can lead to bacterial translocation across the intestine, and increase the level of bacteria throughout the systemic circulation with increased inflammatory status, thus promoting the further development of HF [9]. These studies had significantly increased attention towards the connection between our gut and heart. Thus, recognition of the gut–heart axis may lead to new insights and breakthroughs in the therapies for HF [10, 11].

However, comprehensive analysis of the composition and metabolism of the microbiome in HF has not been conducted. Thus, studies are needed to investigate the fecal microbiome in association with HF and further reveal the effects of fecal metabolic changes in disease pathogenesis. Dahl salt-sensitive rat model is a well-established model of hypertensive heart failure [12–14]. Therefore, in the present study, we performed animal studies using Dahl salt-sensitive rats to evaluate intestinal microbial communities and metabolic profiles of H-HF, using 16S rRNA gene sequencing and metabolomics, to clarify the pathogenesis and consequences of HF.

## Result

### Echocardiographic and blood pressure measurement

**Fig. 1:**
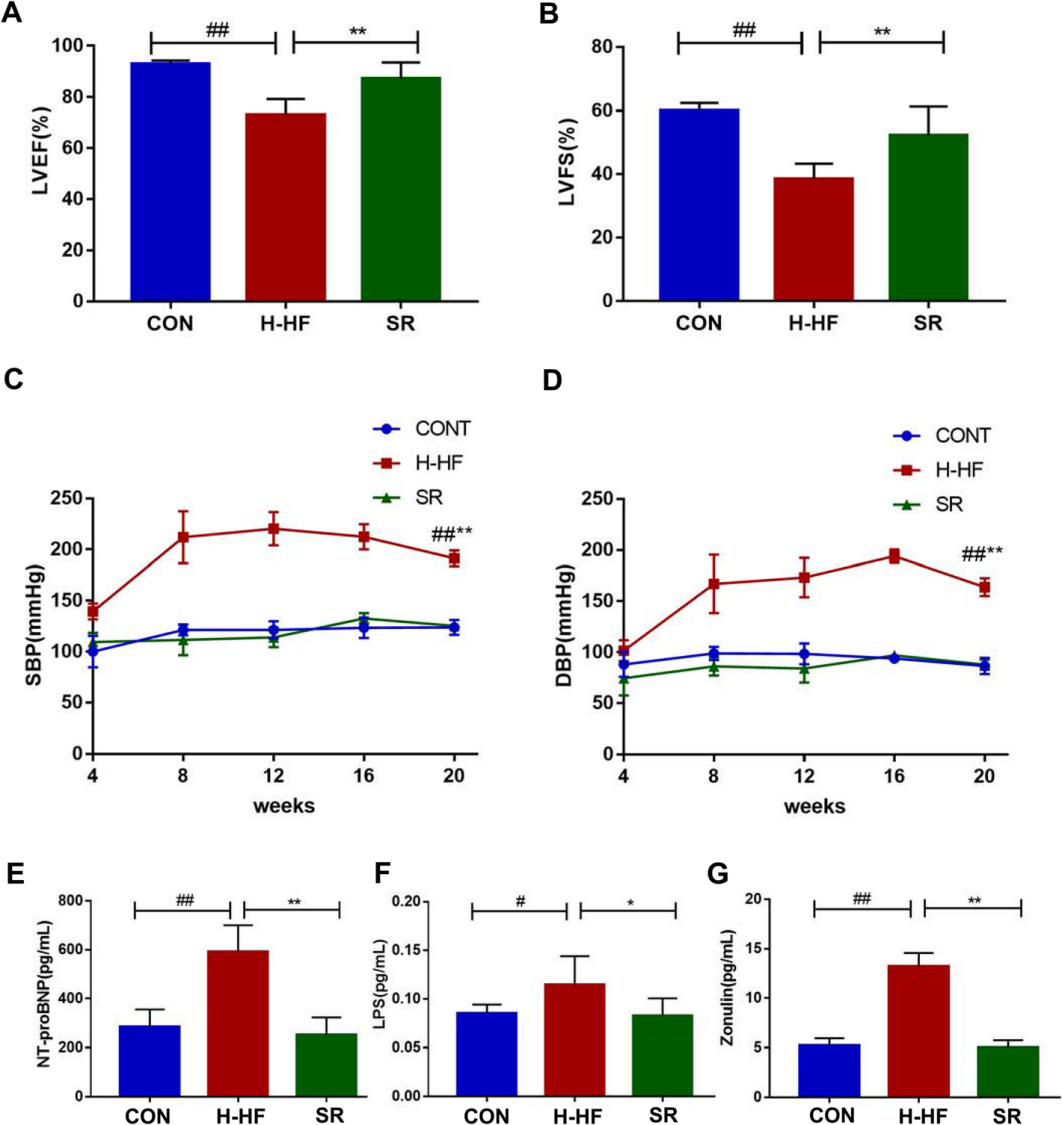
Echocardiographic data on LVEF and LVFS, serum concentration of Nt-proBNP, Zonulin, LPS, and blood pressure. **a**: left ventricular ejection fraction (LVEF); **b**: left ventricular fractional shortening (LVFS). **c**: systolic blood pressure (SBP); **d**: diastolic blood pressure (DBP); **e**: NT-proBNP was assessed by ELISA; **f**: LPS was assessed by ELISA. **g**: Zonulin was assessed by ELISA. ^#^*P* < 0.05, ^##^*P* < 0.01 compared with the CON group; *P < 0.05,** *P* < 0.01 compared with the SR group; Data are presented as the mean ± SD; *n* = 8

The echocardiographic parameters were shown in Fig. 1a and b,compared to the CON group and the SR group, both LVEF and LVFS decreased in H-HF group, suggesting compromised cardiac function, consistent with extant literature [14]. SBP and DBP were significantly higher in H-HF group, whereas blood pressure in the CON group and the SR group did not significantly change over time (Fig. 1c and d).

### Concentration of NT-proBNP, LPS, Zonulin in serum

The N-terminal proB-type natriuretic peptide (NT-proBNP) concentration is a sensitive and reliable biomarker for the diagnosis of heart failure. Our results revealed a significant increase in the NT-proBNP in the H-HF group compared to the CON group and the SR group (Fig. 1e). The intestinal barrier function is crucial for gut homeostasis. Serum lipopolysaccharide (LPS) was measured as an indicator of intestinal barrier function, and Zonulin was considered a marker of intestinal permeability. As shown in Fig. 1f and g, compared to the CON group and the SR group, the LPS and the Zonulin in the H-HF group significantly increased, indicating increased intestinal permeability and compromised intestinal barrier in the H-HF group rats.

### Histological results

**Fig. 2:**
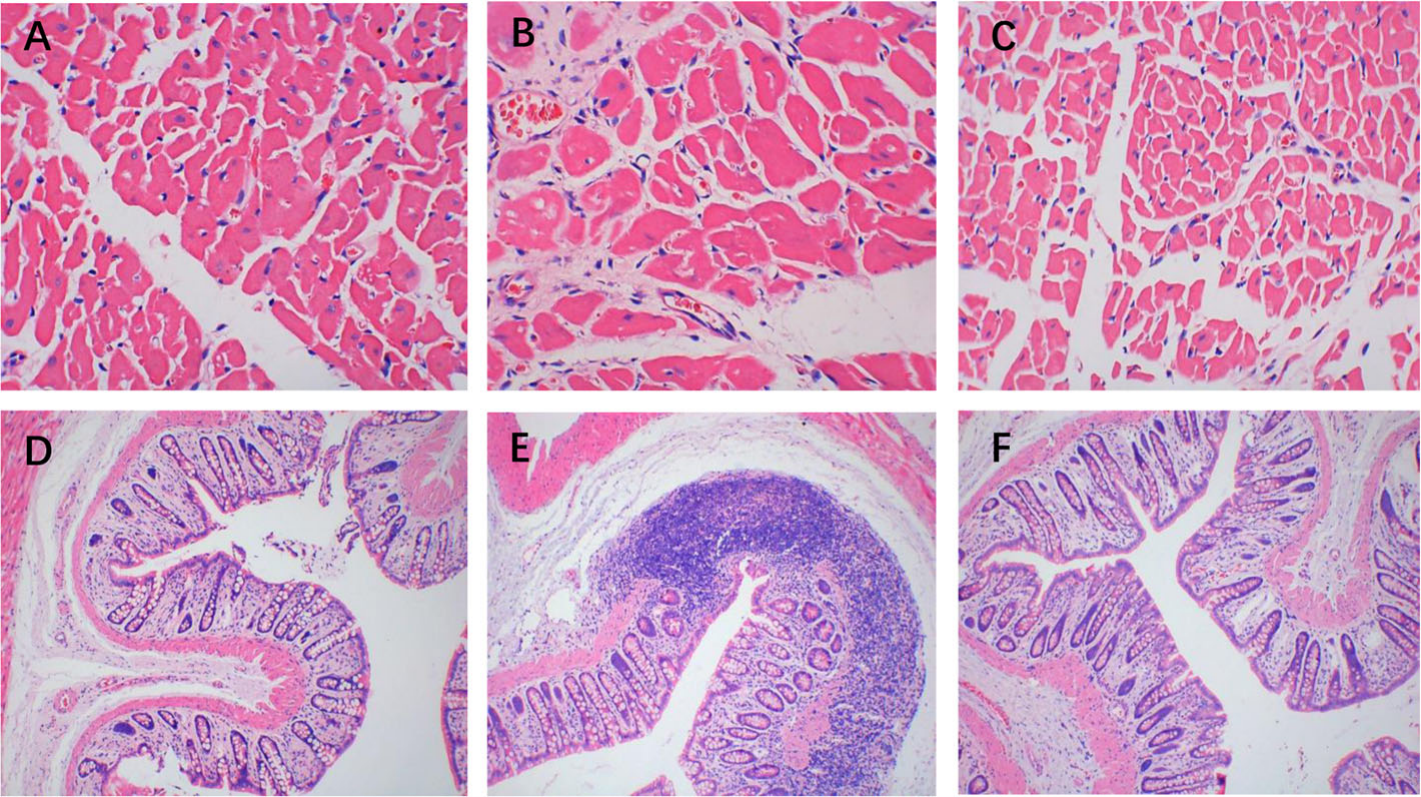
The pathological changes in the heart tissue (**a**, **b**, **c**: × 400) and colon tissues (**d**, **e**, **f**: × 100) in rats (Hematoxylin-eosin (HE) Staining). **a**, **d**: CON group; **b**, **e**: H-HF group; **c**, **f**: SR group

HE staining revealed the distribution of myocardial cells was regular in both CON group and SR group, while the myocardial cells in the H-HF group were swollen with irregular shapes and disordered arrangements, and substantial inflammatory cellular infiltrate in the myocardial interstitium was observed (Fig. 2a-c). HE-stained colonic section of the H-HF group exhibited integrity loss of the intestinal mucosa and infiltration of inflammatory cells into the colon tissue with lymphoid hyperplasia, which was not observed in the CON and SR groups (Fig. 2 d-f). This suggests intestinal barrier damage (leaky gut) in the H-HF group.

### Changes in metabolomics features of H-HF

**Fig. 3:**
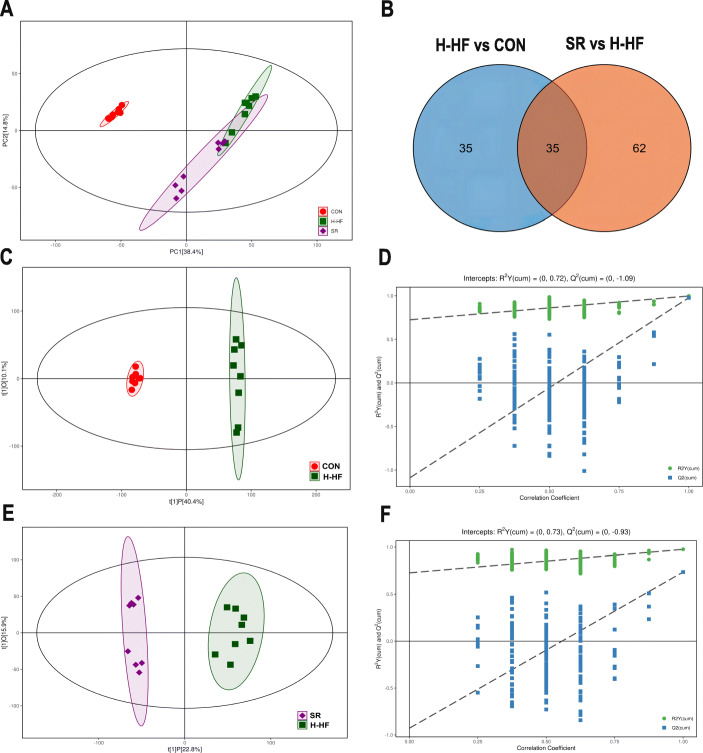
Results of multivariate analysis among three groups. Each dot with three kinds of color (green, H-HF model group; red, CON group; purple, SR group) represented the different sample. **a**: PCA score plots of among three groups (R^2^X = 0.632); **b**: Venn diagram showed the overlapping and unique differential metabolites amongst the comparison groups. **c**: OPLS-DA score plots of CON and H-HF group (R^2^X = 0.505, R^2^Y = 0.997, Q^2^ = 0.977); **d**:The permutation test(*n* = 200) for the OPLS-DA model of CON and H-HF group; **e**: OPLS-DA score plots of H-HF and SR group (R^2^X = 0.387, R^2^Y = 0.976, Q^2^ = 0.736); **f**: The permutation test (*n* = 200) for the OPLS-DA model of H-HF and SR group

PCA was applied to visualize metabolic alterations of the three experimental groups. As shown in the Fig. 3a, the CON group, H-HF group and SR group can be clearly separated. A segregation was visible between the CON and H-HF groups, indicating that certain significant biochemical changes occurred after the high salt diet. In addition, further OPLS-DA analysis also displays that the three groups were obviously separated (Fig. 3c,e). Meanwhile, a statistical validation of the OPLS-DA model was performed using 200 permutation tests, as shown in Fig. 3d and f, the model was reliable.

**Fig. 4:**
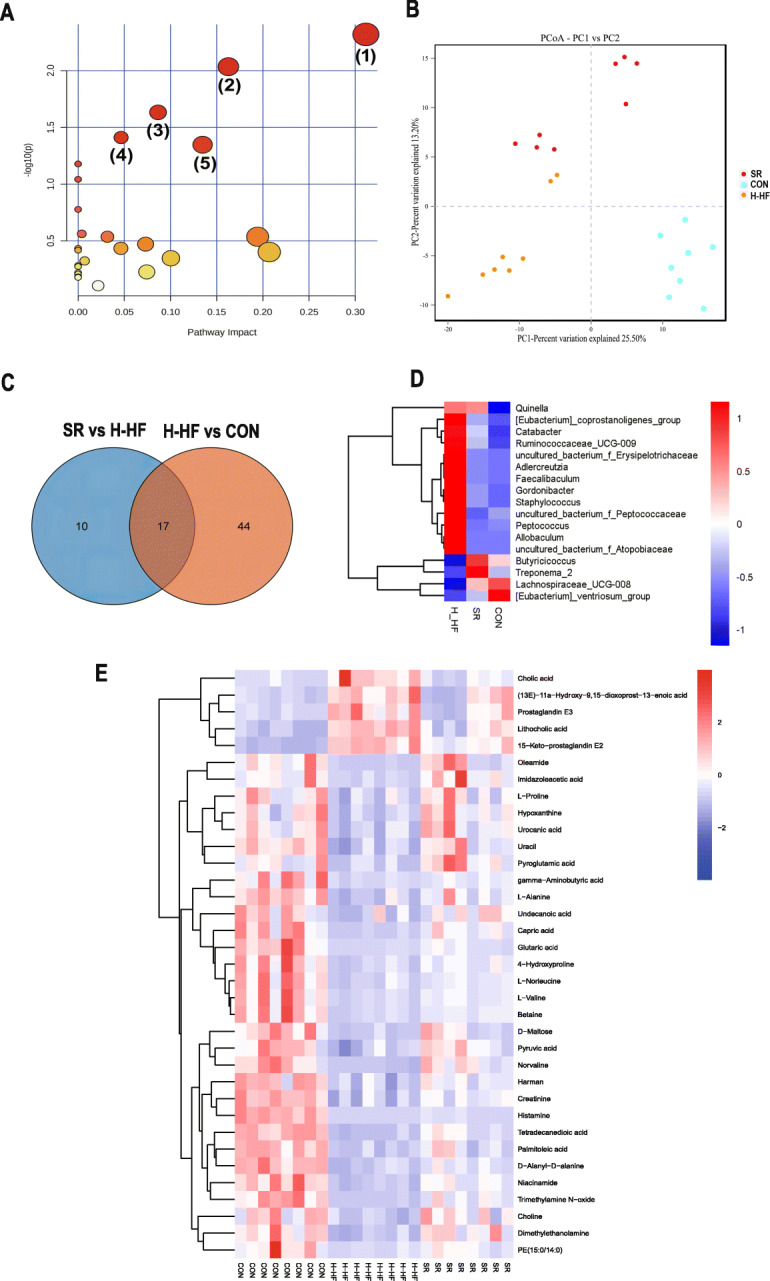
**a**: Summary of the pathway analysis with MetPA of differential metabolites; **b**: PCoA analysis of three groups; **c**: Venn diagrams of three groups; **d**: Heat map of the differential gut microbiota of three groups; **e**: Heat map of the differential metabolites of three groups

The metabolites with VIP > 1.0 and *p* < 0.05 were considered as significantly changed. In comparison of the H-HF group and CON group, 70 metabolites showed significantly different levels (Table S1), and 97 metabolites in the SR group were significantly different from those in the H-HF group (Table S2). The common differential metabolites were analyzed by Venn diagram (Fig. 3b), and it revealed that 35 metabolites were common of the three groups, the results are shown in Table 1. These metabolites could be regarded as potential biomarkers of H-HF. The heatmap of differential metabolite biomarkers is displayed in Fig. 4e. Differentially expressed biomarkers mainly consisted of organonitrogen compounds, organoheterocyclic compounds, organic oxygen compounds, organic nitrogen compounds, organic acids and derivatives, lipids and lipid-like molecules, and alkaloids and derivatives. Among them, studies have shown that metabolites associated with gut microbiota metabolism included creatinine, lithocholic acid, cholic acid, capric acid, glutaric acid, trimethylamine N-oxide, choline, and betaine [15].

**Table 1:** Identification of potential biomarkers of H-HF

No	Retention time(s)	Metabolite identification	Molecular formula	Ion form	MZ	KEGG ID
1	230.706	Pyruvic acid	C_3_H_4_O_3_	ES−	87.00772076	C00022
2	77.84335	Uracil	C_4_H_4_N_2_O_2_	ES−	111.01899	C00106
3	282.828	L-Norleucine	C_6_H_13_NO_2_	ES−	130.0863208	C01933
4	66.92525	Lithocholic acid	C_24_H_40_O_3_	ES−	375.2900014	C03990
5	210.881	Cholic acid	C_24_H_40_O_5_	ES−	407.280768	C00695
6	189.278	Hypoxanthine	C_5_H_4_N_4_O	ES+	137.0456494	C00262
7	324.4045	L-Proline	C_5_H_9_NO_2_	ES−	114.0550451	C00148
8	48.528	Capric acid	C_10_H_20_O_2_	ES−	171.1384219	C01571
9	314.297	L-Valine	C_5_H_11_NO_2_	ES+	118.0862667	C00183
10	288.356	Choline	C_5_H_14_NO	ES+	104.1071126	C00114
11	336.777	gamma-Aminobutyric acid	C_4_H_9_NO_2_	ES−	102.0550772	C00334
12	46.0601	Harman	C_12_H_10_N_2_	ES+	183.091462	C09209
13	190.9455	Creatinine	C_4_H_7_N_3_O	ES+	114.0663089	C00791
14	402.345	D-Maltose	C_12_H_22_O_11_	ES+	365.1042254	C00208
15	47.4337	Undecanoic acid	C_11_H_22_O_2_	ES−	185.1540159	C17715
16	359.129	L-Alanine	C_3_H_7_NO_2_	ES+	90.05519587	C00041
17	398.0985	Glutaric acid	C_5_H_8_O_4_	ES−	131.034148	C00489
18	326.4825	15-Keto-prostaglandin E2	C_20_H_30_O_5_	ES−	349.2016798	C04707
19	223.7705	Oleamide	C_18_H_35_NO	ES+	282.2778295	C19670
20	234.303	Tetradecanedioic acid	C_15_H_24_NO_4_PS	ES−	257.1754713	C11002
21	302.881	Urocanic acid	C_6_H_6_N_2_O_2_	ES+	139.0499338	C00785
22	394.914	Niacinamide	C_6_H_6_N_2_O	ES+	123.0551588	C00153
23	281.996	Dimethylethanolamine	C_9_H_16_NO_8_PR_2_	ES+	90.09161492	C04308
24	255.7855	(13E)-11a-Hydroxy-9,15-dioxoprost-13-enoic acid	C_20_H_32_O_5_	ES−	351.2172613	C04654
25	358.403	4-Hydroxyproline	C_5_H_9_NO_3_	ES−	130.0500239	C01157
26	298.929	Trimethylamine N-oxide	C_3_H_9_NO	ES+	76.07608207	C01104
27	334.166	Betaine	C_5_H_11_NO_2_	ES−	116.0707807	C00719
28	286.6195	Norvaline	C_5_H_11_NO_2_	ES+	118.0862646	C01799
29	43.08045	Palmitoleic acid	C_16_H_30_O_2_	ES−	253.2168827	C08362
30	311.8905	Pyroglutamic acid	C_5_H_7_NO_3_	ES−	128.0344652	C01879
31	212.083	Prostaglandin E3	C_20_H_30_O_5_	ES−	349.2017343	C06439
32	332.553	D-Alanyl-D-alanine	C_6_H_12_N_2_O_3_	ES−	159.0767659	C00993
33	419.462	Histamine	C_5_H_9_N_3_	ES+	112.0869868	C00388
34	174.24	PE(15:0/14:0)	C_7_H_12_NO_8_PR_2_	ES+	650.4736181	C00350
35	333.989	Imidazoleacetic acid	C_5_H_6_N_2_O_2_	ES+	127.050143	C02835

ES + = positive ion mode; ES − = negative ion mode

### Key metabolic pathway analysis for different metabolites

All 35 potential biomarkers were subjected to metabolic pathway analysis (MetPA) using the KEGG online database and MetaboAnalyst. As shown in Fig. 4a, five metabolic pathways were influenced (*P* < 0.05), involving: (1) Histidine metabolism, (2) Arginine and proline metabolism, (3) Alanine, aspartate and glutamate metabolism, (4) Glycine, serine and threonine metabolism, and (5) Glycerophospholipid metabolism. The information for all the affected pathways is described in Table S3.

### Diversity analysis of gut microbiota

**Fig. 5:**
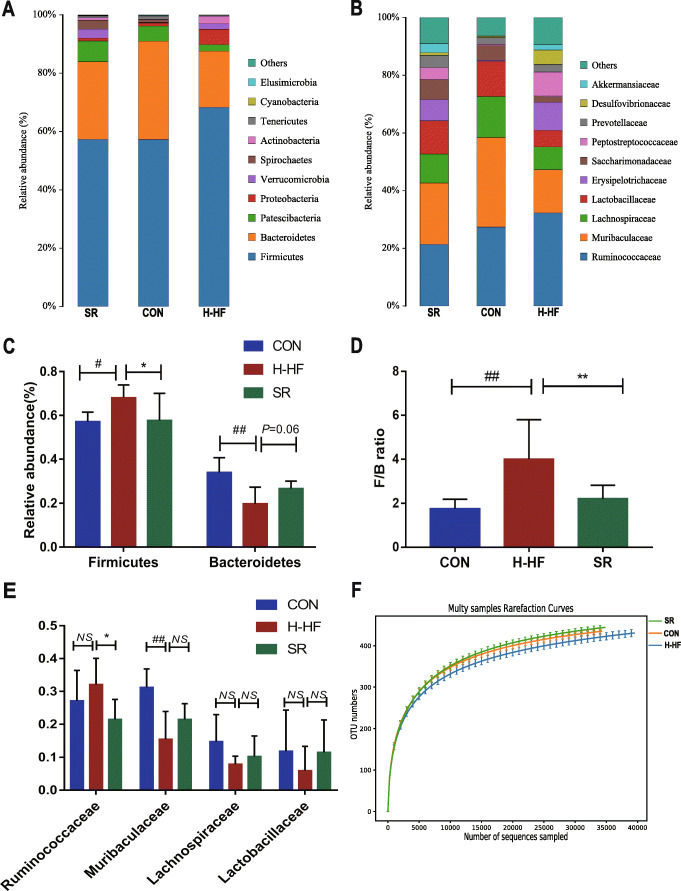
The histogram of species distribution at the phylum (**a**) and family (**b**) levels in three groups; **c**:The relative abundance of *Firmicutes* and *Bacteroidetes*; **d** The F/B ratio; **e**: The relative abundance of *Ruminococcaceae*, *Muribaculaceae*, *Lachnospiraceae*, and *Lactobacillaceae*, **f**: Rarefaction curves;;^#^*P* < 0.05, ^##^*P* < 0.01 compared with the CON group;*P < 0.05, ** *P* < 0.01 compared with the SR group; data are presented as the mean ± SD; *n* = 8

A total of 1,920,409 paired-end reads were obtained from the 24 samples after sequencing (Table S4), with 1,795,548 clean tags after alignment and filtering. Samples contained 74,814 clean tags on average. A total of 609 OTUs (gamma diversity) were obtained at a similarity level of 97%. The community composition of the three groups at the phylum (Fig. 5a) and family (Fig. 5b) was determined. For simplicity, only the 10 most abundant taxa are shown, with all others grouped into “other.”

Compared with the CON group and SR group, the abundance of *Firmicutes* in H-HF group increased significantly (Fig. 5c), while *Bacteroidetes* decreased. Thus, the ratio of *Firmicutes* to *Bacteroidetes* increased significantly (Fig. 5d). An increase in the abundance of *Ruminococcaceae*, contrary to a decrease in the abundance of *Muribaculaceae*, *Lachnospiraceae*, and *Lactobacillaceae*, was observed in the H-HF group compared with the CON group and SR group (Fig. 5e). It revealed that the structure of gut microbiota in H-HF model has changed. Research suggests that the imbalance in the F/B ratio is largely related to energy metabolism [16, 17], an increase in the F/B ratio in our study also indicates the dysregulated energy metabolism, indicating that abnormal energy metabolism was found in H-HF [18], consistent with the existing literature.

Rarefaction curves (Fig. 5f) revealed the amount of sequencing data was sufficient to reflect the true taxonomic diversity. The value of Good’s coverage for each group was higher than 99.8% (Table S4). To investigate the variances of gut microbiota structural diversity in the three groups, Chao1 and Shannon indexes of alpha diversity analysis were used to evaluate richness, evenness and diversity, respectively. Alpha diversity analysis revealed no significant difference in gut microbiota diversity between groups based on Chao 1 and Shannon indices (Fig. S1b,c), but unweighted unifrac PCoA of beta diversity shown that the gut microbiome composition profiles among the SR, CON and H-HF groups were separated (Fig. 4b). ANOSIM tests on UniFrac distance data were applied to permutational multivariate analysis(R = 0.753, *P* < 0.01). That is, the structural diversity of the gut microbiota was significantly different in H-HF group. Our results indicated a significant gut microbial shift during the development of HF, in accord with literatures [19], which highlights the potential role of gut microbiota in HF pathogenesis.

### The alterations of the gut microbiota in H-HF

A total of 61 genera were found to vary significantly different in the H-HF and CON groups (Table S5) and 27 genera were found to differ be significantly different between in the SR and H-HF groups (Table S6). Venn diagram showed the overlapping and unique differential flora amongst the comparison groups. As shown in the Fig. 4c, there were a total of 17 different flora among the three groups. The microflora can be considered as the biomarkers of hypertensive heart failure. Variations in the identified differential gut microbiota of three groups were depicted in the heatmap (Fig. 4d).

Compared to the CON group and SR group, the relative abundance of *[Eubacterium]_coprostanoligenes_group, Catabacter, Ruminococcaceae_UCG-009, uncultured_bacterium_f_Erysipelotrichaceae, Adlercreutzia, Faecalibaculum, Gordonibacter, uncultured_bacterium_f_Peptococcaceae, Allobaculum, Peptococcus, Quinella, Staphylococcus, and uncultured_bacterium_f_Atopobiaceae* increased in the H-HF group, and the abundance of *Butyricicoccus,Treponema_2,Lachnospiraceae_UCG-008,and [Eubacterium]_ventriosum_group* also decreased.

### Correlation of the gut microbiota and fecal metabolic

**Fig. 6:**
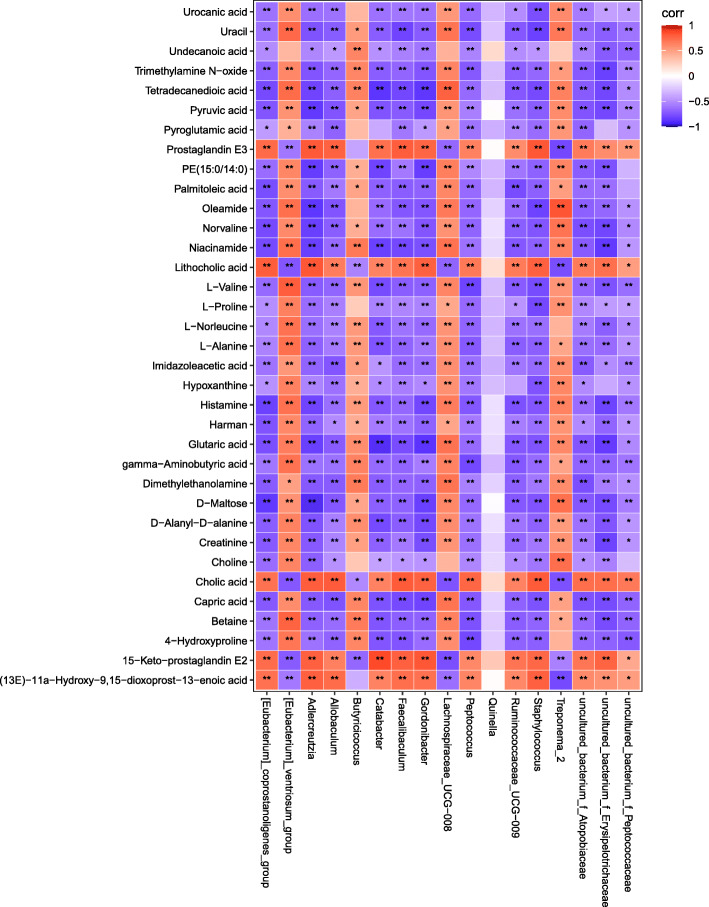
Correlation analysis of relative abundance of gut microbiota in the genus level and fecal metabolite levels. Red means a positive correlation, and blue means a negative correlation. *P < 0.05, ** *P* < 0.01

**Fig. 7:**
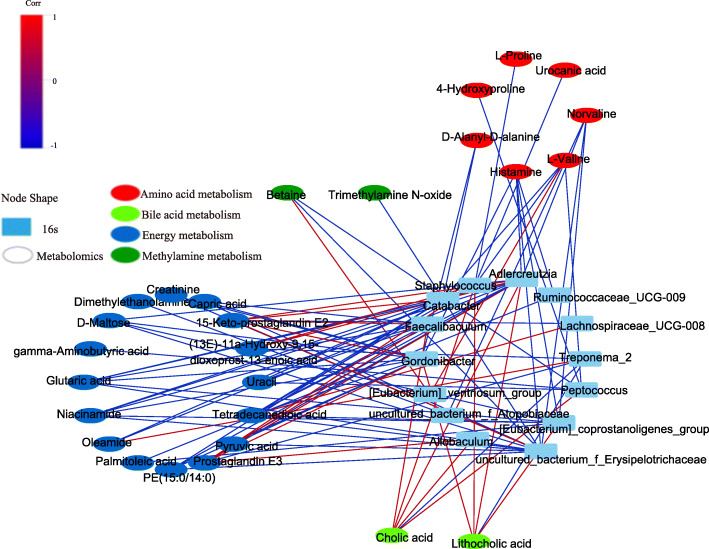
Network diagram of correlation analysis (Pearson’s R-value > 0.75,*P* < 0.05)

Pearson correlation analysis was performed for the screened fecal metabolites and the gut microbiome taxa at genus levels. The significantly related metabolites and gut genera are shown in the form of heat maps (Fig. 6). The related dataset of Pearson’s correlation is described in Table S7. The Pearson’s r-value > 0.75 is considered as a strong correlation. As shown in Fig. 7, for example, Pyruvic acid and D-Maltose were strong negatively related to *Adlercreutzia, Gordonibacter* and *uncultured_bacterium_f_Erysipelotrichaceae*, while Creatinine displayed strong negative correlation with *Gordonibacter,and uncultured_bacterium_f_Erysipelotrichaceae*. Pyruvic acid, D-Maltose and Creatinine are important indicator of energy metabolism. It indicates that *Adlercreutzia, Gordonibacter* and *uncultured_bacterium_f_Erysipelotrichaceae* were closely related to energy metabolism. Betaine correlated positively with *[Eubacterium]_ventriosum_group*, but negatively with *Peptococcus* and *uncultured_bacterium_f_Erysipelotrichaceae*, and a negative correlation was also detected between TMAO and *uncultured_bacterium _f_Erysipelotrichaceae*. Therefore, the gut microbiota plays an important role in methylamine metabolism. TMAO is a metabolite derived from intestinal flora, while choline and betaine are the nutrient precursors of TMAO, they are each independently associated with the prevalence of CVD. Cholic acid and lithocholic acid shown strong negative to *Treponema_2*, but positive to *Staphylococcus, Gordonibacter* and *Adlercreutzia*. Cholic acid is one of the primary bile acids produced from cholesterol in the liver. Therefore, it can be considered H-HF is related to dysbiosis of bile acid metabolism mediated by *treponema_2, staphylococcus, gordonibacter* and *adlercreutzia*.

These correlations indicated that perturbations in the gut microbiome, which may result in a significantly altered metabolomic profile; mainly including energy metabolism, amino acid metabolism, and methylamine metabolism. Our results further confirmed that the variations in gut microbiota and fecal metabolic phenotype associated with the development of HF.

## Discussion

To the best of our knowledge, this is the first examination of specific changes in the gut microbiota composition and function in Dahl salt-sensitive rat model of hypertensive heart failure using both 16S rRNA and LC-MS metabolomics. Altered gut microbial composition and metabolites were observed in the H-HF rat model. The correlation analysis revealed that alterations in GM might contribute to H-HF through amino acid metabolism, bile acid metabolism, methylamine metabolism, energy metabolism and other aspects. The reduction in SCFA-producing bacteria and TMAO might be a notable characteristic for H-HF.

Recent evidence indicates that inflammation drives hypertensive heart failure [20], and impaired epithelial absorption may lead to translocation of microorganisms into the systemic circulation, possibly increasing HF by inducing systemic inflammation [21, 22]. In our study, substantial inflammatory cellular infiltrate in the myocardial interstitium and integrity loss of the intestinal mucosal with infiltration of inflammatory cells into the colon tissue were observed in the H-HF rat model, which indicated inflammation involvement. Increased serum LPS and Zonulin indicated increased intestinal permeability and the loss of gut barrier function, which may lead to the development of metabolic endotoxemia and promote cardiac inflammation, thus exacerbating the development of heart failure.

Different from some clinical studies of GM [23, 24], we found that alpha diversity analysis revealed no significant difference in gut microbiota diversity between groups based on Chao 1 and Shannon indices. However, another clinical study [11] showed the Chao 1 richness and the Shannon index were not significantly different between younger and older patients with HF, which is similar to our results.

To investigate taxonomic changes, we calculated the microbial abundance of the GM at the phylum level and family level. Altered gut microbiota composition was observed in H-HF group compared to CON and SR groups based on 16S rRNA gene sequencing results. The ratio of F/B increased at phylum level, while a decrease in the abundance of *Muribaculaceae*, *Lachnospiraceae*, and *Lactobacillaceae* was shown in the H-HF group compared with the CON group and SR group at family level, which suggested the occurrence of bacterial translocation in H-HF. Butyrate is an energy source for intestinal epithelial cells, modulating the epithelial barrier integrity and playing a local anti-inflammatory role in intestinal mucosa. Therefore, the decrease of butyrate content has been associated with an increase in endotoxemia and inflammation, and the decrease of butyrate-producing bacteria has been associated with inflammatory disease, including diabetes mellitus, obesity, hypertension, and inflammatory bowel disease [25]. Researchers have shown that the *Lachnospiraceae* family, which includes of several butyrate-producing species, is reduced in patients with heart failure [24]. The abundance of *Muribaculaceae* has also been proved to have a strong correlation with propionate and was an important predictor of SCFA concentrations [26]. As SCFA, the energy source for intestinal epithelial cells is also important in maintaining the integrity of intestinal epithelial cells, we could hypothesize that SCFA content is reduced due to the decrease of SCFA producing bacteria, and the imbalance of F/B ratio leads to abnormal energy [16, 17],thus abnormal energy supply may directly affect the systolic function of the heart, which may lead to HF [18], the reduction in SCFA-producing bacteria might be a notable characteristic for H-HF, consistent with the results of clinical studies [27].

Moreover, *Lactobacillus* is essential in the maintenance of intestinal barrier function and integrity [28]. Studies have found that the decrease of *Lactobacillus* seems to promote the development of HF [29]. In our study, a decrease of *Lactobacillus* was also detected in H-HF groups. *Lactobacilli* can reduce cardiac hypertrophy and HF after myocardial infarction and improve left ventricular ejection fraction and shortening fraction, thus regulating intestinal flora may be used to develop a potential therapy to attenuate heart failure [30].

Comparing the different microbiotas between groups at the genus level, there were a total of 17 different flora among the three groups. The microflora can be considered as the biomarkers of hypertensive heart failure. To further assess the impact of a shifted gut microbiome on the host, we conducted metabolome analyses. A series of studies have been published which have highlighted the power of metabolic profiling to expand our understanding of metabolic derangements of the HF [31]. In our study, 35 metabolites have been identified as biomarkers for hypertensive heart failure, and 5 metabolic pathways were influenced, including: Histidine metabolism, Arginine and proline metabolism, Alanine, aspartate and glutamate metabolism, Glycine, serine and threonine metabolism and Glycerophospholipid metabolism. The pathways mainly involved energy metabolism and amino acid metabolism, it indicated that there are abnormities of energy metabolism and amino acid metabolism in H-HF.

Among these differential metabolites, TMAO is considered to be positively associated with cardiovascular disease. TMAO, a metabolites of the gut microbiota from specific dietary nutrients, is linked to a higher risk of HF, and a combination of TMAO and NT-proBNP could provide additional prognostic information [32]. Research shown that elevated plasma TMAO level in patients with HF is associated with poorer prognoses [33]. Systematic review and meta-analysis also demonstrated a positive dose-dependent relationship between TMAO plasma levels and increased cardiovascular risk and mortality [34]. Different from the present studies of TMAO, our study shown that the TMAO content in the feces of H-HF group was lower than the CON group and SR group, results were inconsistent perhaps owing to the different samples examined (e.g., plasma versus feces samples).

Gut microbiota perturbations associated with metabolic phenotype can be used to explore the possible mechanisms in the development of diseases. Peng et al., integrated 16S rRNA sequencing, metagenomics, and metabolomics to characterize gut microbial composition, function, and fecal metabolic phenotype in non-obese Type 2 diabetic goto-kakizaki rats [35], the results suggested that an altered gut microbiota is associated with T2DM pathogenesis. Yu et al. studied the variations in gut microbiota and fecal metabolic phenotype associated with depression by 16S rRNA gene sequencing and LC/MS-based metabolomics [36], and showed a strong correlation between gut microbiota, fecal metabolites, and catecholamine levels. Herein, we observed a significant correlation between gut microbiota at genus level and fecal metabolites through Pearson’s correlation analysis. The correlation indicated that perturbations in the gut microbiome, which may result in a significantly altered amino acid metabolism, bile acid metabolism, TMAO metabolism, energy metabolism and other aspects.

Aberrant energy metabolism is one of the most important signs of HF [37]. Dysfunction of energy metabolism can further promote the development and deterioration of HF. Therefore, an improvement in energy metabolism has been proposed as a potential treatment for HF. Recently, the importance of gut microbiota to the body’s energy metabolism has been widely acknowledged. Our study shows that *Adlercreutzia, Gordonibacter* and *uncultured_bacterium_f_Erysipelotrichaceae* were closely related to energy metabolism. Therefore, regulation of related flora may be a new way to improve host energy metabolism.

Bile acids (BAs) are secreted by the liver and released into the small intestine during a meal where they aid in the absorption of dietary fat and fat-soluble vitamins [38]. BAs have emerged as important mediators of metabolic homeostasis, and have been proposed to play a direct role in regulating cardiovascular physiology and gut dysbiosis-induced HF [39]. BAs have been shown to regulate microbiota both directly and indirectly [40]. Our study shows that *treponema_2, staphylococcus, gordonibacter* and *adlercreutzia* were closely related to bile acid metabolism, which indicates that bile acid is closely related to intestinal flora, which is consistent with the literature.

Our results further confirmed that the variations in gut microbiota and fecal metabolic phenotype were associated with the development of HF, and an intervention to correct gut microbiota composition could be an innovative therapeutic strategy for HF.

## Conclusion

In this study, an integrated approach of 16S rRNA gene sequencing combined with LC-MS based metabolomics was performed to assess the changes variations of gut microbiota in hypertensive heart failure rats. A total of 17 significantly altered bacterial genera and 35 metabolites were identified as the biomarkers of H-HF. Our results showed that HF significantly altered not only the gut microbiota composition but also fecal metabolic phenotype. In addition, correlation analysis revealed that some altered gut microbiota genera were strongly correlated with changed fecal metabolites. The reduction in SCFA-producing bacteria and TMAO might be a notable characteristic for H-HF. Overall, HF may contribute to changes in intestinal flora structure and metabolic function, regulated gut microbiota-related metabolites may be the potential biomarkers for diagnosis, prevention and treatment of HF.

## Methods

### Animals and treatments

Six-week-old Dahl salt-sensitive (SS, *n* = 16) rats and salt-resistant consomic SS.13BN (SR group, *n* = 8**)** rats with a body weight of 200-220 g were purchased from the Beijing Weitong Lihua Animal Co., Ltd., with a qualified number of 1,100,111,911,056,756,. Animals were housed in a specific pathogen-free area with ambient temperature (22 ± 5 °C) and a 12/12 h light/dark cycle. After one week of adaptation, the SS rats were randomly allocated into two groups (8 per group): control (CON) group and hypertensive heart failure (H-HF) model. The CON group was given a low-salt diet containing 0.3% NaCl. To exclude the effects of high salt on the results, the SR group was set. The SR group and H-HF group were given a high-salt diet containing 8% NaCl for 20 weeks [41]. All feed was provided by Beijing Keao Xieli Feed Co., Ltd., (Beijing, China). Food and water were provided ad libitum throughout the experiments. Animal protocols in this study were supervised and approved by the Institutional Animal Care and Use Committee of Hunan University of Chinese Medicine.

### Sample collections and preparation

The rats were sacrificed using urethane anesthesia (1.0 g/kg, i.p.). Fecal samples were collected immediately after defecation at the end of the experiment (20 weeks), and blood samples were collected from the abdominal aorta. All blood samples were processed into serum aliquots on the day of collection and stored at − 80 °C just before the following analysis. Heart tissues were fixed in 4% paraformaldehyde solution, and 5-μm-thick paraffin-embedded tissue sections were cut and stained with hematoxylin and eosin. The serum concentrations of NT-proBNP and LPS were detected using ELISA kits following the manufacturer’s instructions (NT-proBNP Elisa Kit and LPS Elisa Kit: CUSABIO, Wuhan, China, Zonulin Elisa Kit: mlbio, Shanghai, China).

### Echocardiography and blood pressure measurement

Cardiac functions were evaluated before the animals were sacrificed using an echocardiography method. After anesthetizing with urethane (1.0 g/kg, i.p.), all rats underwent echocardiography using a SonoScape-S2N ultrasound system (Shenzhen Kaili technology co., Ltd.). The following parameters were measured from two-dimensional images and M-mode interrogation taken from the parasternal long-axis view at the papillary muscle level. The left ventricular ejection fraction (LVEF) and left ventricular fractional shortening (LVFS) were calculated according to the Teichholtz formula. Blood pressure was measured using a Volume Pressure Recording (VPR) system (CODA; Kent Scientific). For each animal, the systolic blood pressure (SBP) and diastolic blood pressure (DBP) were calculated as the average of 3 independent measurements.

### Fecal metabolomics

Samples comprising 50 mg of fecal was placed in an EP tube, and 1 mL extraction solution (acetonitrile: methanol: water = 2:2:1, with isotopically-labelled internal standard mixture) was added. After 30s vortexing, the samples were homogenized at 35 Hz for 4 min and sonicated for 5 min in ice-water bath. The homogenization and sonication cycle were repeated for 3 times. Then the samples were incubated for 1 h at − 40 °C and centrifuged at 12000 rpm for 15 min at 4 °C. The resulting supernatant was transferred to a fresh glass vial for analysis. The quality control (QC) sample was prepared by mixing an equal aliquot of the supernatants from each samples (Fig. S1a). LC-MS/MS analyses were performed using an UHPLC system (Vanquish, Thermo Fisher Scientific) with a UPLC BEH Amide column (2.1 mm × 100 mm, 1.7 μm) coupled to Q Exactive HFX mass spectrometer (Orbitrap MS, Thermo). The raw data were converted to the mzXML format using ProteoWizard and processed with an in-house program, which was developed using R and based on XCMS, for peak detection, extraction, alignment, and integration. Then an in-house MS2 database (BiotreeDB) was applied for metabolite annotation. The cutoff for annotation was set at 0.3. The methods used in this study were in accordance with the published literature [13, 42]. Data was acquired in positive and negative ion modes, with the two sets of data combined for analysis.

In this study, 13,929 peaks in positive ion mode and 11,910 peaks in negative ion mode were detected, and among them, 1066 metabolites were found in positive ion mode, while 346 metabolites were found in negative ion mode after relative standard deviation de-noising.

The data were trimmed using Compound Discoverer 2.1 (Thermo Fisher Scientific, Waltham, MA, United States) and imported into SIMCA16.0.2 software package (Sartorius Stedim Data Analytics AB, Umea, Sweden) for principle component analysis (PCA) and orthogonal projections to latent structures discriminate analysis (OPLS-DA). Then, a 7-fold cross validation was performed to calculate the value of R^2^ and Q^2^.Furthermore, the value of variable importance in the projection (VIP) of the first principal component in OPLS-DA analysis was obtained. It summarizes the contribution of each variable to the model. The metabolites with VIP > 1.0 and *p* < 0.05 (Student’s t test) were considered as significantly changed. In addition, commercial databases including KEGG (http://www.genome.jp/kegg/) and MetaboAnalyst (http://www.metaboanalyst.ca/) were used for pathway enrichment analysis. The Data analysis method used in this study is consistent with published literature [42].

### 16S rRNA gene sequencing analysis

Total genomic DNA from fecal samples was extracted by Tiangen Fecal Genomic DNA Extraction Kit under the manufacturer’s instruction. The V3-V4 regions of 16S rRNA genes were PCR-amplified using the following primers: 338F: 5′- ACTCCTACGGGAGGCAGCA-3′ and 806R: 5′-GGACTACHVGGGTWTCTAAT-3′, and amplification products were purified, quantified and homogenized to get a sequencing library. Library QC was performed for constructing libraries, qualified libraries were sequenced on Illumina HiSeq 2500. The original image data files obtained by Illumina HiSeq high-throughput sequencing were converted into Sequenced Reads by Base Calling analysis, the results were stored in FASTQ format files. Paired-ends sequences were merged then filtered in three steps: 1) PE reads merge: FLASH v1.2.7 [43] software was used to merge reads through overlap, the obtained merged sequences were Raw Tags; 2) Tags filtering: Trimmomatic v0.33 software was used to filter merged Raw Tags to get high quality Clean Tags;3) Remove Chimera: UCHIME v4.2 software was used [44] to identify and remove chimeric sequences to get Effective Tags.

Sequence analysis was performed by Uparse software (Uparse v7.0.100, http://drive5.com/uparse/) [45]. Sequences with ≥97% similarity were assigned to the same OTU. Representative sequence for each OTU was compared to the Silva Database (http://www.arb-silva.de/) [46] using Mothur (version v.1.30) to identify taxonomic information. In order to study phylogenetic relationships between OTUs, multiple sequence alignment was conducted using the MUSCLE software (Version 3.8.31, http://www.drive5.com/muscle/) [47] . All samples were normalized and mothur software was used to analyze alpha diversity of samples. QIIME software (Version 1.9.1) was used to perform beta diversity analysis. Finally, ANOVA analysis was performed to screen species with significant differences at genus level (*P* < 0.05).

### Statistical analysis

Statistical analysis was performed using IBM SPSS Statistics 22.0 (Chicago, USA). Differences between groups were evaluated by one-way analysis of variance (ANOVA). The relationships between fecal metabolites and the gut microbiome taxa were assessed using Pearson correlation. The significance threshold was set at *P* < 0.05 for all tests.

## Supplementary Information

**Additional file 1: Fig. S1. a**: The PCA score of three group and QC samples; **b**: Shannon index; **c**: Chao1 index; Data are presented as the mean ± SD; *n* = 8.

**Additional file 2: Table S1.** The different fecal metabolites between H-HF and CON group.

**Additional file 3: Table S2.** The different fecal metabolites between H-HF and SR group.

**Additional file 4: Table S3.** The information for all the affected pathways.

**Additional file 5: Table S4.** The sequences information of fecal samples.

**Additional file 6: Table S5.** The different flora between H-HF and CON group.

**Additional file 7: Table S6.** The different flora between H-HF and SR group.

**Additional file 8: Table S7.** The related dataset of Pearson’s correlation.

## Data Availability

The data collected in the present study were properly analyzed and summarized in the Results section. The raw data were deposited in NCBI Sequence Read Archive (SRA) (accession numbers for NCBI: BioProject: PRJNA672260 for 16S rRNA sequencing).

## Abbreviations

GM: gut microbiome
HF: heart failure
H-HF: hypertensive heart failure
SCFA: short-chain fatty acids
TMAO: trimethylamine N-oxide
NT-proBNP: N-terminal proB-type natriuretic peptide
LPS: lipopolysaccharide
PCoA: Principal coordinates analysis
F/B: Firmicutes to Bacteroidetes ratio
OTU: Operational taxonomic unit
PCA: principle component analysis
OPLS-DA: orthogonal projections to latent structures discriminate analysis
VIP: variable importance in the projection
LVEF: left ventricular ejection fraction
LVFS: left ventricular fractional shortening
SBP: systolic blood pressure
DBP: diastolic blood pressure

## References

[1] Bui AL, Horwich TB, Fonarow GC (2011). Epidemiology and risk profile of heart failure. Nat Rev Cardiol.

[2] Ponikowski P, Voors AA, Anker SD, Bueno H, Cleland JGF, Coats AJS, Falk V, González-Juanatey JR, Harjola VP, Jankowska EA, Jessup M, Linde C, Nihoyannopoulos P, Parissis JT, Pieske B, Riley JP, Rosano GMC, Ruilope LM, Ruschitzka F, Rutten FH, van der Meer P, ESC Scientific Document Group (2016). 2016 ESC guidelines for the diagnosis and treatment of acute and chronic heart failure: the task force for the diagnosis and treatment of acute and chronic heart failure of the European Society of Cardiology (ESC) developed with the special contribution of the heart failure association (HFA) of the ESC. Eur Heart J.

[3] Bromfield S, Muntner P (2013). High blood pressure: the leading global burden of disease risk factor and the need for worldwide prevention programs. Curr Hypertens Rep.

[4] Sonnenburg JL, Bäckhed F (2016). Diet-microbiota interactions as moderators of human metabolism. Nature.

[5] Witkowski M, Weeks TL, Hazen SL (2020). Gut microbiota and cardiovascular disease. Circ Res.

[6] Tang WH, Kitai T, Hazen SL (2017). Gut microbiota in cardiovascular health and disease. Circ Res.

[7] Jia Q, Li H, Zhou H, Zhang X, Zhang A, Xie Y, Li Y, Lv S, Zhang J (2019). Role and effective therapeutic target of gut microbiota in heart failure. Cardiovasc Ther.

[8] Krack A, Sharma R, Figulla HR, Anker SD (2005). The importance of the gastrointestinal system in the pathogenesis of heart failure. Eur Heart J.

[9] Tang WHW, Li DY, Hazen SL (2019). Dietary metabolism, the gut microbiome, and heart failure. Nat Rev Cardiol.

[10] Li L, Zhong S, Cheng B, Qiu H, Hu Z (2020). Cross-talk between gut microbiota and the heart: a new target for the herbal medicine treatment of heart failure?. eCAM.

[11] Kamo T, Akazawa H, Suda W, Saga-Kamo A, Shimizu Y, Yagi H, Liu Q, Nomura S, Naito AT, Takeda N, Harada M, Toko H, Kumagai H, Ikeda Y, Takimoto E, Suzuki JI, Honda K, Morita H, Hattori M, Komuro I (2017). Dysbiosis and compositional alterations with aging in the gut microbiota of patients with heart failure. PLoS One.

[12] Yoshida J, Yamamoto K, Mano T, Sakata Y, Nishikawa N, Nishio M, Ohtani T, Miwa T, Hori M, Masuyama T (2004). AT1 receptor blocker added to ACE inhibitor provides benefits AT advanced stage of hypertensive diastolic heart failure. Hypertension.

[13] Akahori H, Tsujino T, Naito Y, Matsumoto M, Sasaki N, Iwasaku T, Eguchi A, Sawada H, Hirotani S, Masuyama T (2014). Atorvastatin ameliorates cardiac fibrosis and improves left ventricular diastolic function in hypertensive diastolic heart failure model rats. J Hypertens.

[14] Klotz S, Hay I, Zhang G, Maurer M, Wang J, Burkhoff D (2006). Development of heart failure in chronic hypertensive Dahl rats. Hypertension.

[15] Nicholson JK, Holmes E, Kinross J, Burcelin R, Gibson G, Jia W, Pettersson S (2012). Host-gut microbiota metabolic interactions. Science (New York, NY).

[16] Chen R, Wang J, Zhan R, Zhang L, Wang X (2019). Fecal metabonomics combined with 16S rRNA gene sequencing to analyze the changes of gut microbiota in rats with kidney-yang deficiency syndrome and the intervention effect of you-gui pill. J Ethnopharmacol.

[17] Turnbaugh PJ, Ley RE, Mahowald MA, Magrini V, Mardis ER, Gordon JI (2006). An obesity-associated gut microbiome with increased capacity for energy harvest. Nature.

[18] Ventura-Clapier R, Garnier A, Veksler V (2004). Energy metabolism in heart failure. J Physiol.

[19] Zheng A, Yi H, Li F, Han L, Yu J, Cheng X, Su H, Hong K, Li J (2019). Changes in gut microbiome structure and function of rats with isoproterenol-induced heart failure. Int Heart J.

[20] Dick SA, Epelman S (2016). Chronic heart failure and inflammation: what do we really know?. Circ Res.

[21] Verbrugge FH, Dupont M, Steels P, Grieten L, Malbrain M, Tang WH, Mullens W (2013). Abdominal contributions to cardiorenal dysfunction in congestive heart failure. J Am Coll Cardiol.

[22] Nagatomo Y, Tang WH (2015). Intersections between microbiome and heart failure: revisiting the gut hypothesis. J Card Fail.

[23] Luedde M, Winkler T, Heinsen FA, Rühlemann MC, Spehlmann ME, Bajrovic A, Lieb W, Franke A, Ott SJ, Frey N (2017). Heart failure is associated with depletion of core intestinal microbiota. ESC Heart Fail.

[24] Kummen M, Mayerhofer CCK, Vestad B, Broch K, Awoyemi A, Storm-Larsen C, Ueland T, Yndestad A, Hov JR, Trøseid M (2018). Gut microbiota signature in heart failure defined from profiling of 2 independent cohorts. J Am Coll Cardiol.

[25] Bach Knudsen KE, Lærke HN, Hedemann MS, Nielsen TS, Ingerslev AK, Gundelund Nielsen DS, Theil PK, Purup S, Hald S, Schioldan AG (2018). Impact of diet-modulated butyrate production on intestinal barrier function and inflammation. Nutrients.

[26] Smith BJ, Miller RA, Ericsson AC, Harrison DC, Strong R, Schmidt TM (2019). Changes in the gut microbiome and fermentation products concurrent with enhanced longevity in acarbose-treated mice. BMC Microbiol.

[27] Chen X, Li HY, Hu XM, Zhang Y, Zhang SY (2019). Current understanding of gut microbiota alterations and related therapeutic intervention strategies in heart failure. Chin Med J.

[28] O'Callaghan J, O'Toole PW (2013). Lactobacillus: host-microbe relationships. Curr Top Microbiol Immunol.

[29] Lai CH, Tsai CC, Kuo WW, Ho TJ, Day CH, Pai PY, Chung LC, Huang CC, Wang HF, Liao PH, Huang CY (2016). Multi-strain probiotics inhibit cardiac myopathies and autophagy to prevent heart injury in high-fat diet-fed rats. Int J Med Sci.

[30] Gan XT, Ettinger G, Huang CX, Burton JP, Haist JV, Rajapurohitam V, Sidaway JE, Martin G, Gloor GB, Swann JR, Reid G, Karmazyn M (2014). Probiotic administration attenuates myocardial hypertrophy and heart failure after myocardial infarction in the rat. Circ Heart Fail.

[31] Turer AT (2013). Using metabolomics to assess myocardial metabolism and energetics in heart failure. J Mol Cell Cardiol.

[32] Suzuki T, Heaney LM, Bhandari SS, Jones DJ, Ng LL (2016). Trimethylamine N-oxide and prognosis in acute heart failure. Heart.

[33] Li W, Huang A, Zhu H, Liu X, Huang X, Huang Y, Cai X, Lu J, Huang Y (2020). Gut microbiota-derived trimethylamine N-oxide is associated with poor prognosis in patients with heart failure. Med J Aust.

[34] Schiattarella GG, Sannino A, Toscano E, Giugliano G, Gargiulo G, Franzone A, Trimarco B, Esposito G, Perrino C (2017). Gut microbe-generated metabolite trimethylamine-N-oxide as cardiovascular risk biomarker: a systematic review and dose-response meta-analysis. Eur Heart J.

[35] Peng W, Huang J, Yang J, Zhang Z, Yu R, Fayyaz S, Zhang S, Qin YH (2019). Integrated 16S rRNA sequencing, Metagenomics, and metabolomics to characterize gut microbial composition, function, and fecal metabolic phenotype in non-obese type 2 diabetic Goto-Kakizaki rats. Front Microbiol.

[36] Yu M, Jia H, Zhou C, Yang Y, Zhao Y, Yang M, Zou Z (2017). Variations in gut microbiota and fecal metabolic phenotype associated with depression by 16S rRNA gene sequencing and LC/MS-based metabolomics. J Pharm Biomed Anal.

[37] Stanley WC, Recchia FA, Lopaschuk GD (2005). Myocardial substrate metabolism in the normal and failing heart. Physiol Rev.

[38] Branchereau M, Burcelin R, Heymes C (2019). The gut microbiome and heart failure: a better gut for a better heart. Rev Endocr Metab Disord.

[39] Vasavan T, Ferraro E, Ibrahim E, Dixon P, Gorelik J, Williamson C (2018). Heart and bile acids - Clinical consequences of altered bile acid metabolism. Biochim Biophys Acta Mol Basis Dis.

[40] Ridlon JM, Harris SC, Bhowmik S, Kang DJ, Hylemon PB (2016). Consequences of bile salt biotransformations by intestinal bacteria. Gut Microbes.

[41] Igreja B, Pires NM, Wright LC, Soares-da-Silva P (2019). Effects of zamicastat treatment in a genetic model of salt-sensitive hypertension and heart failure. Eur J Pharmacol.

[42] Liu T, Gu X, Li LX, Li M, Li B, Cui X, Zuo XL (2020). Microbial and metabolomic profiles in correlation with depression and anxiety co-morbidities in diarrhoea-predominant IBS patients. BMC Microbiol.

[43] Magoc T, Salzberg SL (2011). FLASH: fast length adjustment of short reads to improve genome assemblies. Bioinformatics.

[44] Edgar RC, Haas BJ, Clemente JC, Quince C, Knight R (2011). UCHIME improves sensitivity and speed of chimera detection. Bioinformatics.

[45] Edgar RC (2013). UPARSE: highly accurate OTU sequences from microbial amplicon reads. Nat Methods.

[46] Quast C, Pruesse E, Yilmaz P, Gerken J, Schweer T, Yarza P, Peplies J, Glöckner FO (2013). The SILVA ribosomal RNA gene database project: improved data processing and web-based tools. Nucleic Acids Res.

[47] Edgar RC (2004). MUSCLE: multiple sequence alignment with high accuracy and high throughput. Nucleic Acids Res.

