# Supplementary material for: Changes of gut microbiome composition and metabolites associated with hypertensive heart failure rats
Source: BMC Microbiol. 2021 May 5;21:141. doi: 10.1186/s12866-021-02202-5 (PMC8097775; doi:10.1186/s12866-021-02202-5)
Supplement: Supplementary file 2 — Additional file 2: Table S1. The different fecal metabolites between H-HF and CON group. [file 12866_2021_2202_MOESM2_ESM.docx]

**Changes of Gut Microbiome Composition and Metabolites Associated with Hypertensive Heart Failure Rats**

Lin Li ^1,2^, Sen-jie Zhong ^3^, Si-yuan Hu ^1^,Bin Cheng ^3^, Hong Qiu ^3^, Zhi-xi Hu ^1,2^*****

1. The Domestic First-class Discipline Construction Project of Chinese Medicine, Hunan University of Chinese Medicine, Changsha, Hunan, China
2. Institute of Traditional Chinese Medicine Diagnostics, Hunan University of Chinese Medicine, Changsha, Hunan, China
3. Post-Graduate School, Hunan University of Chinese Medicine, Changsha,Hunan, China

*****Correspondence should be addressed to Zhixi Hu: 003405@hnucm.edu.cn

Table S1: The different fecal metabolites between H-HF and CON group.

| No | Ion form | name | rt | mz | MEAN（H-HF） | MEAN（CON） | VIP | P-VALUE | Q-VALUE |
| --- | --- | --- | --- | --- | --- | --- | --- | --- | --- |
|  | ES− | o-Cresol | 111.3470 | 107.0493 | 0.000015 | 0.000048 | 1.1242 | 0.0103 | 0.0055 |
|  | ES− | Pyruvic acid | 230.7060 | 87.0077 | 0.000016 | 0.000026 | 1.2088 | 0.0005 | 0.0006 |
|  | ES− | Sarcosine | 359.3650 | 88.0394 | 0.000167 | 0.000522 | 1.2494 | 0.0002 | 0.0003 |
|  | ES− | Uracil | 77.8434 | 111.0190 | 0.003886 | 0.010679 | 1.3396 | 0.0000 | 0.0000 |
|  | ES− | L-Norleucine | 282.8280 | 130.0863 | 0.000527 | 0.002590 | 1.2719 | 0.0035 | 0.0024 |
|  | ES− | Lithocholic acid | 66.9253 | 375.2900 | 0.006837 | 0.001564 | 1.4707 | 0.0000 | 0.0000 |
|  | ES− | Cholic acid | 210.8810 | 407.2808 | 0.000760 | 0.000162 | 1.4114 | 0.0042 | 0.0028 |
|  | ES+ | Hypoxanthine | 189.2780 | 137.0456 | 0.001857 | 0.004207 | 1.0091 | 0.0129 | 0.0065 |
|  | ES+ | Asacoumarin A | 444.1210 | 399.2117 | 0.000004 | 0.000001 | 1.3461 | 0.0031 | 0.0022 |
|  | ES− | L-Proline | 324.4045 | 114.0550 | 0.000090 | 0.000194 | 1.0719 | 0.0059 | 0.0036 |
|  | ES− | Capric acid | 48.5280 | 171.1384 | 0.000025 | 0.000229 | 1.3731 | 0.0033 | 0.0023 |
|  | ES+ | L-Valine | 314.2970 | 118.0863 | 0.000233 | 0.001379 | 1.3168 | 0.0049 | 0.0031 |
|  | ES+ | Choline | 288.3560 | 104.1071 | 0.000916 | 0.001911 | 1.1987 | 0.0036 | 0.0025 |
|  | ES+ | Genistein | 25.8799 | 271.0592 | 0.000000 | 0.000005 | 1.1733 | 0.0007 | 0.0007 |
|  | ES− | Succinic acid | 398.7660 | 117.0183 | 0.000359 | 0.002059 | 1.1573 | 0.0182 | 0.0086 |
|  | ES− | (R)-3-Hydroxybutyric acid | 236.1290 | 103.0391 | 0.000031 | 0.000372 | 1.2876 | 0.0099 | 0.0053 |
|  | ES− | gamma-Aminobutyric acid | 336.7770 | 102.0551 | 0.000011 | 0.000115 | 1.2935 | 0.0086 | 0.0048 |
|  | ES+ | Deoxyguanosine | 177.2650 | 268.1032 | 0.000249 | 0.000461 | 1.1369 | 0.0073 | 0.0042 |
|  | ES− | Chenodeoxycholic acid | 158.1760 | 391.2853 | 0.058308 | 0.017715 | 1.2655 | 0.0005 | 0.0005 |
|  | ES+ | 4-Aminobutyraldehyde | 363.6870 | 88.0760 | 0.000024 | 0.000063 | 1.2655 | 0.0035 | 0.0024 |
|  | ES− | Isobutyric acid | 94.5809 | 87.0441 | 0.001456 | 0.010073 | 1.2754 | 0.0219 | 0.0100 |
|  | ES− | Xanthine | 225.3540 | 151.0252 | 0.000683 | 0.001758 | 1.1426 | 0.0027 | 0.0020 |
|  | ES− | 3-Hydroxybutyric acid | 150.0635 | 103.0391 | 0.000005 | 0.000033 | 1.0744 | 0.0007 | 0.0007 |
|  | ES+ | Harman | 46.0601 | 183.0915 | 0.000106 | 0.000276 | 1.2226 | 0.0000 | 0.0001 |
|  | ES+ | Creatinine | 190.9455 | 114.0663 | 0.000046 | 0.000095 | 1.3697 | 0.0000 | 0.0000 |
|  | ES+ | D-Maltose | 402.3450 | 365.1042 | 0.000003 | 0.000030 | 1.3718 | 0.0011 | 0.0010 |
|  | ES+ | 3-Formyl-6-hydroxyindole | 59.1776 | 162.0546 | 0.000095 | 0.001664 | 1.4153 | 0.0029 | 0.0021 |
|  | ES− | Undecanoic acid | 47.4337 | 185.1540 | 0.000037 | 0.000087 | 1.0498 | 0.0092 | 0.0050 |
|  | ES+ | L-Alanine | 359.1290 | 90.0552 | 0.000092 | 0.000310 | 1.3134 | 0.0000 | 0.0001 |
|  | ES− | Glutaric acid | 398.0985 | 131.0341 | 0.000047 | 0.000579 | 1.4799 | 0.0019 | 0.0016 |
|  | ES− | 15-Keto-prostaglandin E2 | 326.4825 | 349.2017 | 0.000010 | 0.000002 | 1.5444 | 0.0000 | 0.0000 |
|  | ES+ | 2-Propene-1-thiol | 175.9880 | 75.0267 | 0.000077 | 0.000019 | 1.3732 | 0.0000 | 0.0000 |
|  | ES+ | Oleamide | 223.7705 | 282.2778 | 0.000000 | 0.000003 | 1.4862 | 0.0058 | 0.0035 |
|  | ES+ | Taurine | 309.5390 | 126.0219 | 0.000014 | 0.000040 | 1.2155 | 0.0081 | 0.0046 |
|  | ES− | Glycocholic acid | 271.0920 | 464.3016 | 0.000091 | 0.000000 | 1.4593 | 0.0232 | 0.0105 |
|  | ES+ | Iprobenfos | 376.2270 | 289.1009 | 0.000008 | 0.000017 | 1.3926 | 0.0000 | 0.0000 |
|  | ES+ | 3-(1,1-Dimethyl-2-propenyl)-8-(3-methyl-2-butenyl)xanthyletin | 416.1680 | 365.2055 | 0.000007 | 0.000001 | 1.5090 | 0.0000 | 0.0001 |
|  | ES+ | Acetylsalvipisone | 443.4880 | 355.1852 | 0.000008 | 0.000002 | 1.4330 | 0.0000 | 0.0001 |
|  | ES− | 2-Hydroxybutyric acid | 195.0030 | 103.0391 | 0.000105 | 0.000879 | 1.3898 | 0.0000 | 0.0000 |
|  | ES+ | Kanzonol F | 427.8675 | 421.1959 | 0.000004 | 0.000000 | 1.4591 | 0.0000 | 0.0001 |
|  | ES− | 20-Carboxy-leukotriene B4 | 352.1915 | 365.1966 | 0.000019 | 0.000011 | 1.2998 | 0.0001 | 0.0001 |
|  | ES− | Tetradecanedioic acid | 234.3030 | 257.1755 | 0.000013 | 0.000190 | 1.4272 | 0.0000 | 0.0000 |
|  | ES+ | Urocanic acid | 302.8810 | 139.0499 | 0.000205 | 0.000483 | 1.1369 | 0.0023 | 0.0018 |
|  | ES− | Tridecanoic acid | 44.3343 | 213.1853 | 0.000137 | 0.000286 | 1.1488 | 0.0019 | 0.0015 |
|  | ES+ | L-Acetylcarnitine | 321.2995 | 204.1227 | 0.000007 | 0.000016 | 1.0500 | 0.0012 | 0.0011 |
|  | ES− | Pentadecanoic acid | 226.2950 | 241.2170 | 0.000042 | 0.000076 | 1.1673 | 0.0073 | 0.0042 |
|  | ES+ | Niacinamide | 394.9140 | 123.0552 | 0.000013 | 0.000060 | 1.4782 | 0.0002 | 0.0003 |
|  | ES+ | 1-Methyladenine | 252.2480 | 150.0773 | 0.000011 | 0.000044 | 1.2387 | 0.0155 | 0.0076 |
|  | ES+ | Dimethylethanolamine | 281.9960 | 90.0916 | 0.000009 | 0.000025 | 1.3664 | 0.0007 | 0.0007 |
|  | ES− | Methylsuccinic acid | 366.2130 | 131.0342 | 0.000021 | 0.000094 | 1.3474 | 0.0000 | 0.0001 |
|  | ES+ | Daidzein | 46.0363 | 255.0644 | 0.000002 | 0.000128 | 1.3508 | 0.0020 | 0.0016 |
|  | ES− | (13E)-11a-Hydroxy-9,15-dioxoprost-13-enoic acid | 255.7855 | 351.2173 | 0.000055 | 0.000022 | 1.4157 | 0.0006 | 0.0007 |
|  | ES− | 4-Hydroxyproline | 358.4030 | 130.0500 | 0.000005 | 0.000014 | 1.2585 | 0.0042 | 0.0028 |
|  | ES+ | Valyl-Phenylalanine | 284.5095 | 265.1534 | 0.000013 | 0.000006 | 1.2476 | 0.0049 | 0.0031 |
|  | ES+ | Propionylcarnitine | 276.2040 | 218.1382 | 0.000005 | 0.000008 | 1.1249 | 0.0006 | 0.0006 |
|  | ES+ | Boviquinone 4 | 103.5065 | 413.2648 | 0.000094 | 0.000041 | 1.0063 | 0.0087 | 0.0048 |
|  | ES+ | 1-Kestose | 463.7790 | 527.1579 | 0.000000 | 0.000001 | 1.2236 | 0.0036 | 0.0025 |
|  | ES+ | Trimethylamine N-oxide | 298.9290 | 76.0761 | 0.000003 | 0.000018 | 1.4886 | 0.0004 | 0.0005 |
|  | ES+ | 1-Methylhistamine | 358.0670 | 126.1025 | 0.000010 | 0.000334 | 1.4865 | 0.0016 | 0.0013 |
|  | ES+ | Methylpyrazine | 319.4205 | 95.0606 | 0.000028 | 0.000098 | 1.4933 | 0.0000 | 0.0000 |
|  | ES− | Betaine | 334.1660 | 116.0708 | 0.000013 | 0.000071 | 1.3344 | 0.0036 | 0.0025 |
|  | ES+ | N,O-Didesmethylvenlafaxine | 287.2310 | 250.1791 | 0.000279 | 0.000051 | 1.5097 | 0.0002 | 0.0003 |
|  | ES+ | Norvaline | 286.6195 | 118.0863 | 0.000052 | 0.000263 | 1.4216 | 0.0011 | 0.0010 |
|  | ES− | Palmitoleic acid | 43.0805 | 253.2169 | 0.000113 | 0.000528 | 1.4605 | 0.0000 | 0.0000 |
|  | ES− | Pyroglutamic acid | 311.8905 | 128.0345 | 0.000081 | 0.000158 | 1.0243 | 0.0267 | 0.0117 |
|  | ES− | Prostaglandin E3 | 212.0830 | 349.2017 | 0.000343 | 0.000125 | 1.3209 | 0.0025 | 0.0019 |
|  | ES− | D-Alanyl-D-alanine | 332.5530 | 159.0768 | 0.000020 | 0.000100 | 1.4285 | 0.0000 | 0.0000 |
|  | ES+ | Histamine | 419.4620 | 112.0870 | 0.000008 | 0.000121 | 1.5510 | 0.0000 | 0.0000 |
|  | ES+ | PE(15:0/14:0) | 174.2400 | 650.4736 | 0.000007 | 0.000038 | 1.2796 | 0.0340 | 0.0140 |
|  | ES+ | Imidazoleacetic acid | 333.9890 | 127.0501 | 0.000004 | 0.000012 | 1.2618 | 0.0216 | 0.0099 |

ES+ = positive ion mode; ES− = negative ion mode.
