# Supplementary material for: Changes of gut microbiome composition and metabolites associated with hypertensive heart failure rats
Source: BMC Microbiol. 2021 May 5;21:141. doi: 10.1186/s12866-021-02202-5 (PMC8097775; doi:10.1186/s12866-021-02202-5)
Supplement: Supplementary file 3 — Additional file 3: Table S2. The different fecal metabolites between H-HF and SR group. [file 12866_2021_2202_MOESM3_ESM.docx]

**Changes of Gut Microbiome Composition and Metabolites Associated with Hypertensive Heart Failure Rats**

Lin Li ^1,2^, Sen-jie Zhong ^3^, Si-yuan Hu ^1^,Bin Cheng ^3^, Hong Qiu ^3^, Zhi-xi Hu ^1,2^*****

1. The Domestic First-class Discipline Construction Project of Chinese Medicine, Hunan University of Chinese Medicine, Changsha, Hunan, China
2. Institute of Traditional Chinese Medicine Diagnostics, Hunan University of Chinese Medicine, Changsha, Hunan, China
3. Post-Graduate School, Hunan University of Chinese Medicine, Changsha,Hunan, China

*****Correspondence should be addressed to Zhixi Hu: 003405@hnucm.edu.cn

Table S2: The different fecal metabolites between H-HF and SR group

| No | Ion form | name | rt | mz | MEAN(H-HF） | MEAN(SR) | VIP | P-VALUE | Q-VALUE |
| --- | --- | --- | --- | --- | --- | --- | --- | --- | --- |
|  | neg4 | Uracil | 77.843350 | 111.018990 | 0.003886 | 0.008350 | 1.581641 | 0.006129 | 0.018675 |
|  | neg2 | Pyruvic acid | 230.706000 | 87.007721 | 0.000016 | 0.000023 | 1.447451 | 0.003389 | 0.015729 |
|  | neg5 | Linoelaidic acid | 40.491100 | 279.232230 | 0.001086 | 0.020153 | 1.749610 | 0.014365 | 0.028340 |
|  | neg9 | L-Norleucine | 282.828000 | 130.086321 | 0.000527 | 0.000922 | 1.482628 | 0.017419 | 0.031474 |
|  | neg10 | Caprylic acid | 49.289300 | 143.106977 | 0.000088 | 0.000141 | 1.477062 | 0.005362 | 0.017567 |
|  | neg12 | Lithocholic acid | 66.925250 | 375.290001 | 0.006837 | 0.003593 | 1.240093 | 0.001366 | 0.011447 |
|  | neg13 | p-Cresol | 47.388400 | 107.049281 | 0.000003 | 0.000011 | 1.483644 | 0.020355 | 0.034242 |
|  | pos3 | Pyrrolidine | 296.217000 | 72.081191 | 0.000192 | 0.000351 | 1.676354 | 0.003763 | 0.016180 |
|  | neg14 | Isohyodeoxycholic  acid | 178.396000 | 391.285219 | 0.008308 | 0.004203 | 1.098404 | 0.036050 | 0.046733 |
|  | pos4 | 1-Pyrroline | 344.076000 | 70.065550 | 0.000008 | 0.000017 | 1.545603 | 0.015790 | 0.029780 |
|  | neg15 | Cholic acid | 210.881000 | 407.280768 | 0.000760 | 0.000217 | 1.566016 | 0.006795 | 0.019585 |
|  | pos5 | Hypoxanthine | 189.278000 | 137.045649 | 0.001857 | 0.004440 | 1.647844 | 0.006625 | 0.019362 |
|  | pos6 | Isoquinoline | 36.385200 | 130.065051 | 0.000017 | 0.000046 | 1.723498 | 0.000776 | 0.009722 |
|  | neg16 | L-Proline | 324.404500 | 114.055045 | 0.000090 | 0.000177 | 1.266828 | 0.027327 | 0.039986 |
|  | neg17 | Capric acid | 48.528000 | 171.138422 | 0.000025 | 0.000112 | 1.820871 | 0.007074 | 0.019991 |
|  | neg18 | Tauroursodeoxycholic  acid | 168.857000 | 498.290615 | 0.000610 | 0.000188 | 1.471291 | 0.037249 | 0.047601 |
|  | pos8 | L-Valine | 314.297000 | 118.086267 | 0.000233 | 0.000495 | 1.676727 | 0.000523 | 0.008459 |
|  | neg20 | Hydrocinnamic acid | 84.775900 | 149.059844 | 0.000980 | 0.006984 | 1.560323 | 0.011992 | 0.025962 |
|  | pos9 | Piperidine | 283.445000 | 86.096710 | 0.000213 | 0.000416 | 1.699328 | 0.000664 | 0.009211 |
|  | neg21 | 2-Hydroxystearic acid | 37.779100 | 299.259353 | 0.000611 | 0.001830 | 1.658767 | 0.002754 | 0.014771 |
|  | pos13 | Diethanolamine | 255.372500 | 74.096815 | 0.000039 | 0.000064 | 1.361889 | 0.008431 | 0.021774 |
|  | pos14 | Quinoline-4,8-diol | 186.590000 | 162.054708 | 0.000020 | 0.000031 | 1.416269 | 0.022611 | 0.036146 |
|  | pos15 | Choline | 288.356000 | 104.107113 | 0.000916 | 0.001684 | 1.268053 | 0.011645 | 0.025595 |
|  | neg24 | Glyceraldehyde | 148.754000 | 89.023442 | 0.000147 | 0.000284 | 1.752322 | 0.000228 | 0.006237 |
|  | neg26 | 9,10-epoxyoctadecanoic acid | 51.990050 | 297.242992 | 0.000445 | 0.001432 | 1.159657 | 0.036742 | 0.047237 |
|  | neg27 | gamma-Aminobutyric acid | 336.777000 | 102.055077 | 0.000011 | 0.000036 | 1.697027 | 0.002343 | 0.013975 |
|  | pos20 | (Â±)-erythro-Isoleucine | 285.005000 | 132.101744 | 0.000505 | 0.000894 | 1.390353 | 0.021650 | 0.035358 |
|  | neg28 | Leucinic acid | 129.346500 | 131.070459 | 0.000126 | 0.000736 | 1.723317 | 0.029222 | 0.041458 |
|  | pos25 | D-Proline | 492.373000 | 116.070630 | 0.000006 | 0.000009 | 1.521712 | 0.004162 | 0.016594 |
|  | pos26 | Leukoaminochrome | 293.459000 | 152.070369 | 0.000043 | 0.000185 | 1.676958 | 0.002972 | 0.015132 |
|  | neg30 | Benzoic acid | 115.291000 | 121.028606 | 0.000120 | 0.000218 | 1.549332 | 0.002714 | 0.014701 |
|  | neg31 | L-Lysine | 536.224000 | 145.097414 | 0.000006 | 0.000022 | 1.112726 | 0.009621 | 0.023239 |
|  | pos29 | Harman | 46.060100 | 183.091462 | 0.000106 | 0.000159 | 1.102627 | 0.017633 | 0.031689 |
|  | pos31 | 6,7-Dihydro-5-methyl-5H-cyclopenta[b]pyrazine | 253.129500 | 135.091556 | 0.000011 | 0.000017 | 1.208584 | 0.023105 | 0.036537 |
|  | pos32 | Creatinine | 190.945500 | 114.066309 | 0.000046 | 0.000063 | 1.306309 | 0.008195 | 0.021480 |
|  | pos33 | D-Maltose | 402.345000 | 365.104225 | 0.000003 | 0.000016 | 1.357074 | 0.038147 | 0.048236 |
|  | neg38 | Undecanoic acid | 47.433700 | 185.154016 | 0.000037 | 0.000072 | 1.396366 | 0.025716 | 0.038722 |
|  | pos38 | Butyramide | 200.655500 | 88.076034 | 0.000033 | 0.000042 | 1.432448 | 0.003768 | 0.016185 |
|  | pos39 | L-Alanine | 359.129000 | 90.055196 | 0.000092 | 0.000185 | 1.405332 | 0.039618 | 0.049248 |
|  | neg39 | Dodecanedioic acid | 245.043000 | 229.143965 | 0.000320 | 0.000631 | 1.520441 | 0.004001 | 0.016434 |
|  | pos40 | Phenylacetaldehyde | 240.900500 | 121.064846 | 0.000021 | 0.000064 | 1.537900 | 0.017642 | 0.031697 |
|  | pos41 | (-)-Matairesinol 4'-[apiosyl-(1->2)-glucoside] | 359.008000 | 653.241766 | 0.000000 | 0.000000 | 1.383263 | 0.005323 | 0.017516 |
|  | neg42 | Glutaric acid | 398.098500 | 131.034148 | 0.000047 | 0.000140 | 1.567411 | 0.017013 | 0.031062 |
|  | pos43 | 2-Methyl-3-(2-methylpropyl)pyrazine | 145.218500 | 151.122688 | 0.000012 | 0.000030 | 1.323116 | 0.029771 | 0.041907 |
|  | neg43 | Dodecanoic acid | 38.008200 | 199.169739 | 0.000026 | 0.000095 | 1.574855 | 0.038912 | 0.048766 |
|  | neg44 | 15-Keto-prostaglandin E2 | 326.482500 | 349.201680 | 0.000010 | 0.000006 | 1.555880 | 0.002599 | 0.014490 |
|  | pos48 | Oleamide | 223.770500 | 282.277829 | 0.000000 | 0.000003 | 1.534866 | 0.031121 | 0.042984 |
|  | pos51 | N-Ethylglycine | 329.319000 | 104.070694 | 0.000015 | 0.000051 | 1.479347 | 0.041389 | 0.050420 |
|  | pos55 | 3-Methylcytosine | 200.423000 | 126.066069 | 0.000010 | 0.000024 | 1.561694 | 0.008213 | 0.021502 |
|  | neg48 | 8-Hydroxyguanosine | 20.026850 | 298.081555 | 0.000000 | 0.000000 | 1.962386 | 0.002391 | 0.014078 |
|  | pos60 | 3-Methyladenine | 119.607500 | 150.077239 | 0.000044 | 0.000105 | 1.569710 | 0.005320 | 0.017514 |
|  | neg51 | Diacetone alcohol | 67.857750 | 115.075438 | 0.001296 | 0.007178 | 1.275671 | 0.009413 | 0.022997 |
|  | neg52 | Mesalazine | 324.656000 | 152.034580 | 0.000006 | 0.000012 | 1.480262 | 0.007908 | 0.021119 |
|  | neg55 | Tetradecanedioic acid | 234.303000 | 257.175471 | 0.000013 | 0.000075 | 1.752686 | 0.002410 | 0.014118 |
|  | pos66 | Pteroyltriglutamic acid | 465.284000 | 700.224410 | 0.000000 | 0.000000 | 1.383263 | 0.005323 | 0.017516 |
|  | pos68 | Urocanic acid | 302.881000 | 139.049934 | 0.000205 | 0.000479 | 1.457308 | 0.018789 | 0.032806 |
|  | neg58 | (Â±)-2-(1-Methylpropyl)-4,6-dinitrophenol | 57.203500 | 239.059436 | 0.000042 | 0.000024 | 1.238288 | 0.021884 | 0.035553 |
|  | neg60 | 4-Dodecylbenzenesulfonic Acid | 7.063720 | 325.183508 | 0.000007 | 0.000008 | 1.276407 | 0.012906 | 0.026907 |
|  | neg61 | Pyrocatechol | 36.777900 | 109.028588 | 0.000009 | 0.000029 | 1.195278 | 0.029315 | 0.041535 |
|  | neg62 | cis-Vaccenic acid | 40.487300 | 281.247733 | 0.003582 | 0.017887 | 1.723401 | 0.010372 | 0.024146 |
|  | pos72 | 3-Ethylpyridine | 239.338000 | 108.080861 | 0.000019 | 0.000010 | 1.493835 | 0.004760 | 0.017112 |
|  | pos73 | Niacinamide | 394.914000 | 123.055159 | 0.000013 | 0.000031 | 1.857506 | 0.000069 | 0.004390 |
|  | pos75 | Dimethylethanolamine | 281.996000 | 90.091615 | 0.000009 | 0.000022 | 1.729742 | 0.002516 | 0.014331 |
|  | pos78 | 6-Methylquinoline | 216.581000 | 144.080533 | 0.000273 | 0.001834 | 1.788817 | 0.001146 | 0.010900 |
|  | neg67 | (13E)-11a-Hydroxy-9,15-dioxoprost-13-enoic acid | 255.785500 | 351.217261 | 0.000055 | 0.000034 | 1.134983 | 0.048720 | 0.055392 |
|  | pos80 | Beta-Carboline | 45.517650 | 169.075885 | 0.000056 | 0.000197 | 1.789451 | 0.000988 | 0.010460 |
|  | pos81 | Isohumbertiol | 217.906000 | 219.173860 | 0.000035 | 0.000097 | 1.728922 | 0.000045 | 0.003794 |
|  | neg71 | 4-Hydroxyproline | 358.403000 | 130.050024 | 0.000005 | 0.000007 | 1.550666 | 0.005607 | 0.017917 |
|  | pos83 | Creatine | 362.862000 | 132.076528 | 0.000061 | 0.000122 | 1.324476 | 0.040452 | 0.049806 |
|  | neg73 | 9,10-DHOME | 64.363900 | 313.238075 | 0.000142 | 0.000605 | 1.528613 | 0.009546 | 0.023153 |
|  | pos84 | Cinnzeylanol | 281.218500 | 385.218649 | 0.000004 | 0.000011 | 1.712164 | 0.001061 | 0.010669 |
|  | pos86 | beta-Sinensal | 84.225600 | 219.173759 | 0.000016 | 0.000026 | 1.302445 | 0.021125 | 0.034914 |
|  | pos87 | Tryptamine | 216.434000 | 161.107103 | 0.000055 | 0.000386 | 1.792636 | 0.001007 | 0.010514 |
|  | neg74 | Corymboside | 329.097500 | 563.141904 | 0.000000 | 0.000000 | 1.383263 | 0.005323 | 0.017516 |
|  | pos92 | Fragransin C1 | 463.298000 | 375.175494 | 0.000005 | 0.000010 | 1.367095 | 0.009426 | 0.023012 |
|  | pos93 | LysoPE(15:0/0:0) | 225.811500 | 440.276099 | 0.000129 | 0.000650 | 1.526796 | 0.011568 | 0.025512 |
|  | pos94 | LysoPE(16:0/0:0) | 223.978000 | 454.292823 | 0.000048 | 0.000523 | 1.618892 | 0.020391 | 0.034274 |
|  | neg77 | Medicagenic acid | 207.044000 | 501.322021 | 0.000011 | 0.000005 | 1.644095 | 0.000052 | 0.003912 |
|  | pos100 | (alpha-D-mannosyl)7-beta-D-mannosyl-diacetylchitobiosyl-L-asparagine, isoform A (protein) | 380.202000 | 90.055197 | 0.000021 | 0.000044 | 1.214129 | 0.025168 | 0.038275 |
|  | neg79 | 9-OxoODE | 47.522200 | 293.212373 | 0.000023 | 0.000258 | 1.583188 | 0.042236 | 0.050972 |
|  | pos104 | Trimethylamine N-oxide | 298.929000 | 76.076082 | 0.000003 | 0.000008 | 1.663330 | 0.012557 | 0.026554 |
|  | neg80 | 20-Hydroxy-PGE2 | 273.649500 | 367.212615 | 0.000008 | 0.000006 | 1.083265 | 0.042387 | 0.051072 |
|  | neg82 | 24-Epibrassinolide | 198.205000 | 479.337244 | 0.000060 | 0.000034 | 1.323146 | 0.008805 | 0.022255 |
|  | pos108 | 4-Guanidinobutanoic acid | 375.333500 | 146.092191 | 0.000151 | 0.000530 | 1.351159 | 0.036203 | 0.046845 |
|  | pos110 | Columbaridione | 446.903500 | 345.164389 | 0.000011 | 0.000022 | 1.489412 | 0.001653 | 0.012163 |
|  | neg84 | Betaine | 334.166000 | 116.070781 | 0.000013 | 0.000025 | 1.730365 | 0.000242 | 0.006330 |
|  | neg85 | Ethyl dodecanoate | 44.408000 | 227.201012 | 0.000581 | 0.001585 | 1.662410 | 0.007030 | 0.019927 |
|  | pos111 | Riboflavin | 230.431000 | 377.144853 | 0.000016 | 0.000022 | 1.348161 | 0.035485 | 0.046315 |
|  | neg88 | Ecgonine | 219.991000 | 184.097303 | 0.000026 | 0.000011 | 1.307518 | 0.013204 | 0.027205 |
|  | pos134 | Norvaline | 286.619500 | 118.086265 | 0.000052 | 0.000183 | 1.682757 | 0.004508 | 0.016907 |
|  | neg97 | Palmitoleic acid | 43.080450 | 253.216883 | 0.000113 | 0.000253 | 1.091777 | 0.048001 | 0.054901 |
|  | neg99 | Pyroglutamic acid | 311.890500 | 128.034465 | 0.000081 | 0.000221 | 1.655508 | 0.006475 | 0.019160 |
|  | neg100 | Prostaglandin E3 | 212.083000 | 349.201734 | 0.000343 | 0.000181 | 1.257798 | 0.024944 | 0.038090 |
|  | neg105 | D-Alanyl-D-alanine | 332.553000 | 159.076766 | 0.000020 | 0.000040 | 1.507015 | 0.009661 | 0.023285 |
|  | pos173 | Histamine | 419.462000 | 112.086987 | 0.000008 | 0.000016 | 1.421248 | 0.012406 | 0.026398 |
|  | pos610 | PE(15:0/14:0) | 174.240000 | 650.473618 | 0.000007 | 0.000017 | 1.460763 | 0.015413 | 0.029392 |
|  | pos743 | Imidazoleacetic acid | 333.989000 | 127.050143 | 0.000004 | 0.000015 | 1.667016 | 0.025712 | 0.038718 |

ES+ = positive ion mode; ES− = negative ion mode.
