# Supplementary material for: Changes of gut microbiome composition and metabolites associated with hypertensive heart failure rats
Source: BMC Microbiol. 2021 May 5;21:141. doi: 10.1186/s12866-021-02202-5 (PMC8097775; doi:10.1186/s12866-021-02202-5)
Supplement: Supplementary file 4 — Additional file 4: Table S3. The information for all the affected pathways. [file 12866_2021_2202_MOESM4_ESM.docx]

**Changes of Gut Microbiome Composition and Metabolites Associated with Hypertensive Heart Failure Rats**

Lin Li ^1,2^, Sen-jie Zhong ^3^, Si-yuan Hu ^1^,Bin Cheng ^3^, Hong Qiu ^3^, Zhi-xi Hu ^1,2^*****

1. The Domestic First-class Discipline Construction Project of Chinese Medicine, Hunan University of Chinese Medicine, Changsha, Hunan, China
2. Institute of Traditional Chinese Medicine Diagnostics, Hunan University of Chinese Medicine, Changsha, Hunan, China
3. Post-Graduate School, Hunan University of Chinese Medicine, Changsha,Hunan, China

*****Correspondence should be addressed to Zhixi Hu: 003405@hnucm.edu.cn

Table S3: The information for all the affected pathways.

| Pathways | Total | Expected | Hits | Raw p | Impact |
| --- | --- | --- | --- | --- | --- |
| Histidine metabolism | 16 | 0.3605 | 3 | 0.004793 | 0.31147 |
| Arginine and proline metabolism | 38 | 0.8562 | 4 | 0.009224 | 0.16264 |
| Alanine, aspartate and glutamate metabolism | 28 | 0.63088 | 3 | 0.023308 | 0.08654 |
| Glycine, serine and threonine metabolism | 34 | 0.76607 | 3 | 0.03884 | 0.04655 |
| Glycerophospholipid metabolism | 36 | 0.81113 | 3 | 0.044951 | 0.13477 |
| Pantothenate and CoA biosynthesis | 19 | 0.4281 | 2 | 0.06634 | 0 |
| Aminoacyl-tRNA biosynthesis | 48 | 1.0815 | 3 | 0.090758 | 0 |
| Valine, leucine and isoleucine biosynthesis | 8 | 0.18025 | 1 | 0.16702 | 0 |
| Glycosylphosphatidylinositol (GPI)-anchor biosynthesis | 14 | 0.31544 | 1 | 0.27418 | 0.00399 |
| Butanoate metabolism | 15 | 0.33797 | 1 | 0.29068 | 0.03175 |
| Nicotinate and nicotinamide metabolism | 15 | 0.33797 | 1 | 0.29068 | 0.1943 |
| Starch and sucrose metabolism | 18 | 0.40557 | 1 | 0.33805 | 0.07306 |
| Selenocompound metabolism | 20 | 0.45063 | 1 | 0.3679 | 0 |
| Citrate cycle (TCA cycle) | 20 | 0.45063 | 1 | 0.3679 | 0.04634 |
| beta-Alanine metabolism | 21 | 0.47316 | 1 | 0.38234 | 0 |
| Pyruvate metabolism | 22 | 0.49569 | 1 | 0.39645 | 0.20684 |
| Glycolysis / Gluconeogenesis | 26 | 0.58582 | 1 | 0.44984 | 0.10044 |
| Glutathione metabolism | 28 | 0.63088 | 1 | 0.47478 | 0.00709 |
| Glyoxylate and dicarboxylate metabolism | 32 | 0.72101 | 1 | 0.52142 | 0 |
| Cysteine and methionine metabolism | 33 | 0.74354 | 1 | 0.53244 | 0 |
| Pyrimidine metabolism | 39 | 0.87873 | 1 | 0.59355 | 0.0743 |
| Valine, leucine and isoleucine degradation | 40 | 0.90126 | 1 | 0.60295 | 0 |
| Tyrosine metabolism | 42 | 0.94632 | 1 | 0.62112 | 0 |
| Primary bile acid biosynthesis | 46 | 1.0364 | 1 | 0.65508 | 0 |
| Fatty acid biosynthesis | 47 | 1.059 | 1 | 0.6631 | 0 |
| Purine metabolism | 66 | 1.4871 | 1 | 0.78516 | 0.02167 |
