# Supplementary material for: Changes of gut microbiome composition and metabolites associated with hypertensive heart failure rats
Source: BMC Microbiol. 2021 May 5;21:141. doi: 10.1186/s12866-021-02202-5 (PMC8097775; doi:10.1186/s12866-021-02202-5)
Supplement: Supplementary file 6 — Additional file 6: Table S5. The different flora between H-HF and CON group. [file 12866_2021_2202_MOESM6_ESM.docx]

**Changes of Gut Microbiome Composition and Metabolites Associated with Hypertensive Heart Failure Rats**

Lin Li ^1,2^, Sen-jie Zhong ^3^, Si-yuan Hu ^1^,Bin Cheng ^3^, Hong Qiu ^3^, Zhi-xi Hu ^1,2^*****

1. The Domestic First-class Discipline Construction Project of Chinese Medicine, Hunan University of Chinese Medicine, Changsha, Hunan, China
2. Institute of Traditional Chinese Medicine Diagnostics, Hunan University of Chinese Medicine, Changsha, Hunan, China
3. Post-Graduate School, Hunan University of Chinese Medicine, Changsha,Hunan, China

*****Correspondence should be addressed to Zhixi Hu: 003405@hnucm.edu.cn

Table S5: The different flora between H-HF and CON group.

| No | genus | Mean(Cont) | Mean(H-HF) | P value | Q value |
| --- | --- | --- | --- | --- | --- |
|  | uncultured_bacterium_f_Desulfovibrionaceae | 0.001260 | 0.013100 | 0.0190 | 0.0433 |
|  | uncultured_bacterium_f_Peptococcaceae | 0.000527 | 0.001880 | 0.0160 | 0.0370 |
|  | Lachnospiraceae_FCS020_group | 0.000100 | 0.000006 | 0.0130 | 0.0306 |
|  | Parabacteroides | 0.000370 | 0.002480 | 0.0120 | 0.0287 |
|  | Pygmaiobacter | 0.000921 | 0.000102 | 0.0120 | 0.0287 |
|  | Ruminiclostridium_5 | 0.002460 | 0.006290 | 0.0120 | 0.0287 |
|  | Clostridium_sensu_stricto_1 | 0.003270 | 0.018700 | 0.0110 | 0.0277 |
|  | DNF00809 | 0.000096 | 0.000744 | 0.0090 | 0.0243 |
|  | Defluviitaleaceae_UCG-011 | 0.000176 | 0.000483 | 0.0080 | 0.0220 |
|  | Lachnoclostridium_5 | 0.000043 | 0.000006 | 0.0080 | 0.0220 |
|  | Bifidobacterium | 0.000065 | 0.000605 | 0.0070 | 0.0200 |
|  | Ruminococcaceae_NK4A214_group | 0.011000 | 0.032500 | 0.0070 | 0.0200 |
|  | uncultured_bacterium_f_Ruminococcaceae | 0.020800 | 0.074500 | 0.0070 | 0.0200 |
|  | Family_XIII_AD3011_group | 0.000363 | 0.001050 | 0.0060 | 0.0182 |
|  | [Bacteroides]_pectinophilus_group | 0.001630 | 0.000040 | 0.0060 | 0.0182 |
|  | Desulfovibrio | 0.002590 | 0.032200 | 0.0050 | 0.0159 |
|  | [Eubacterium]_xylanophilum_group | 0.005620 | 0.001580 | 0.0050 | 0.0159 |
|  | uncultured_bacterium_f_Eggerthellaceae | 0.000018 | 0.000440 | 0.0040 | 0.0133 |
|  | [Eubacterium]_brachy_group | 0.000154 | 0.000443 | 0.0030 | 0.0102 |
|  | [Eubacterium]_nodatum_group | 0.000364 | 0.001990 | 0.0030 | 0.0102 |
|  | Dubosiella | 0.000199 | 0.037500 | 0.0020 | 0.0071 |
|  | Enterorhabdus | 0.000272 | 0.002630 | 0.0020 | 0.0071 |
|  | Gordonibacter | 0.000164 | 0.001400 | 0.0020 | 0.0071 |
|  | Lachnospiraceae_NK4B4_group | 0.000330 | 0.000086 | 0.0020 | 0.0071 |
|  | Ruminiclostridium_6 | 0.002900 | 0.000305 | 0.0020 | 0.0071 |
|  | Ruminococcaceae_UCG-011 | 0.000094 | 0.001200 | 0.0020 | 0.0071 |
|  | [Ruminococcus]_gauvreauii_group | 0.001240 | 0.000042 | 0.0020 | 0.0071 |
|  | uncultured_bacterium_f_Muribaculaceae | 0.312000 | 0.154000 | 0.0020 | 0.0071 |
|  | Adlercreutzia | 0.000529 | 0.007160 | 0.0010 | 0.0044 |
|  | Allobaculum | 0.000100 | 0.031200 | 0.0010 | 0.0044 |
|  | Butyricicoccus | 0.000275 | 0.000024 | 0.0010 | 0.0044 |
|  | Catabacter | 0.000003 | 0.000540 | 0.0010 | 0.0044 |
|  | Coriobacteriaceae_UCG-002 | 0.000000 | 0.001460 | 0.0010 | 0.0044 |
|  | Corynebacterium_1 | 0.000241 | 0.006920 | 0.0010 | 0.0044 |
|  | Elusimicrobium | 0.001920 | 0.000021 | 0.0010 | 0.0044 |
|  | Facklamia | 0.000003 | 0.000221 | 0.0010 | 0.0044 |
|  | Faecalibaculum | 0.000288 | 0.012100 | 0.0010 | 0.0044 |
|  | GCA-900066225 | 0.000000 | 0.000165 | 0.0010 | 0.0044 |
|  | Jeotgalicoccus | 0.000019 | 0.001430 | 0.0010 | 0.0044 |
|  | Lachnospiraceae_NK4A136_group | 0.084900 | 0.017900 | 0.0010 | 0.0044 |
|  | Lachnospiraceae_UCG-008 | 0.000327 | 0.000031 | 0.0010 | 0.0044 |
|  | Lactococcus | 0.004180 | 0.000021 | 0.0010 | 0.0044 |
|  | Negativibacillus | 0.000000 | 0.000210 | 0.0010 | 0.0044 |
|  | Parvibacter | 0.000032 | 0.000242 | 0.0010 | 0.0044 |
|  | Peptococcus | 0.000062 | 0.000599 | 0.0010 | 0.0044 |
|  | Prevotellaceae_UCG-001 | 0.006130 | 0.000392 | 0.0010 | 0.0044 |
|  | Quinella | 0.000000 | 0.000163 | 0.0010 | 0.0044 |
|  | Romboutsia | 0.004400 | 0.081900 | 0.0010 | 0.0044 |
|  | Ruminococcaceae_UCG-009 | 0.000324 | 0.001160 | 0.0010 | 0.0044 |
|  | Ruminococcaceae_UCG-010 | 0.000392 | 0.002410 | 0.0010 | 0.0044 |
|  | Staphylococcus | 0.000045 | 0.000477 | 0.0010 | 0.0044 |
|  | Subdoligranulum | 0.000003 | 0.000697 | 0.0010 | 0.0044 |
|  | Treponema_2 | 0.008310 | 0.000014 | 0.0010 | 0.0044 |
|  | Tyzzerella | 0.000299 | 0.002220 | 0.0010 | 0.0044 |
|  | UBA1819 | 0.000083 | 0.002950 | 0.0010 | 0.0044 |
|  | [Eubacterium]_coprostanoligenes_group | 0.011900 | 0.071400 | 0.0010 | 0.0044 |
|  | [Eubacterium]_oxidoreducens_group | 0.000868 | 0.000039 | 0.0010 | 0.0044 |
|  | [Eubacterium]_ventriosum_group | 0.000285 | 0.000000 | 0.0010 | 0.0044 |
|  | uncultured_bacterium_f_Atopobiaceae | 0.000032 | 0.000960 | 0.0010 | 0.0044 |
|  | uncultured_bacterium_f_Barnesiellaceae | 0.000000 | 0.000047 | 0.0010 | 0.0044 |
|  | uncultured_bacterium_f_Erysipelotrichaceae | 0.000137 | 0.002540 | 0.0010 | 0.0044 |
